# Sustainable Hydrogen Production, a Review of Methods, Types, Applications, Challenges, and Future Perspectives

**DOI:** 10.1002/gch2.202500086

**Published:** 2025-04-30

**Authors:** Sajid Ali Ansari, Mir Waqas Alam, Neetu Dhanda, Misbah Sehar Abbasi, Mais Emad Ahmed, Alanoud bader alrashidi, Amani Mubarak Al‐Farhan, Buzuayehu Abebe

**Affiliations:** ^1^ Department of Physics College of Science King Faisal University Al‐Ahsa 31982 Saudi Arabia; ^2^ Department of Physics Amity University Haryana Gurugram Haryana 122413 India; ^3^ College of Materials Science and Opto‐Electronic Technology Center of Materials Science and Optoelectronics Engineering CAS Center for Excellence in Topological Quantum Computation CAS Key Laboratory of Vacuum Physics University of Chinese Academy of Sciences Beijing 100049 China; ^4^ Department of Biology College of Sciences University of Baghdad Baghdad 10071 Iraq; ^5^ Department of Applied Chemistry School of Applied Natural Sciences Adama Science and Technology University P.O. Box: 1888 Adama Ethiopia

**Keywords:** biomass conversion, green hydrogen production, hydrogen, offshore, water splitting

## Abstract

Hydrogen is promising as an innovative energy vector beyond its conventional role and receiving international identification as a feasible fuel source. This review provides a concise examination of current advances in hydrogen production techniques employing renewable and conventional energy sources, as well as important difficulties in hydrogen production. Wind and solar are the two most promising sustainable energy sources for hydrogen manufacturing. The hydrogen production from renewable sources generated from undeveloped or other waste flows increases the affordability and flexibility of semi‐centralized and distributed reforming while emitting no net or fewer greenhouse gases. Water electrolysis apparatus powered by wind energy or off‐grid solar can also used in distant places away from the framework. Every hydrogen‐producing technology presents technological obstacles. These obstacles include conversion efficiency, feedstock type, and the requirement to safely integrate the production of hydrogen systems with storage and purification technology.

## Graphical abstract

This graphical abstract illustrates sustainable hydrogen production methods integrating solar, wind, and biomass sources. It highlights electrolysis as the central green hydrogen pathway and contrasts renewable and industrial uses. Color‐coded hydrogen types depict the diversity in production technologies, emphasizing clean energy transitions and future infrastructure for hydrogen as a key energy carrier.

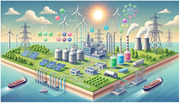

## 1 Introduction

**Figure 1:**
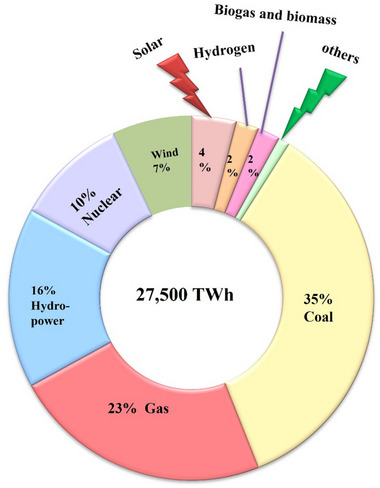
The global proportion of energy sources in 2021.

**Figure 2:**
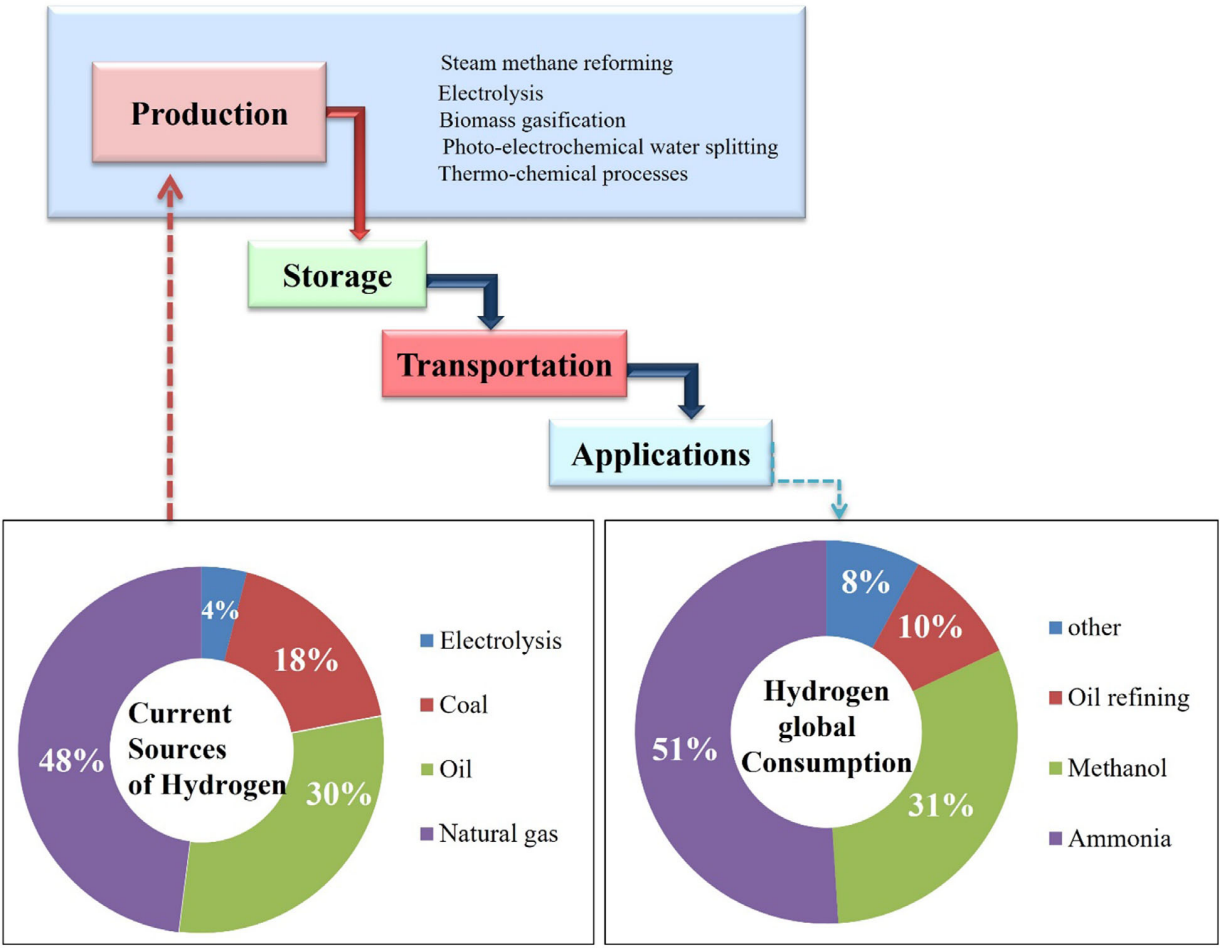
Stages in the hydrogen industry.

In the twenty‐first century, we face a challenge: fulfilling rising global energy demand while mitigating the harmful effects of environmental change.^[^ ^1^ ^]^ The utilization of fossil fuels‐related energy systems, vital for development, has been proved to be unsustainable because of their environmental implications and limited availability.^[^ ^2^, ^3^, ^4^ ^]^ As the industrialized revolution, dependence on fossil fuel leads to a major increase in greenhouse gases and CO_₂_, contributing considerably to global warming.^[^ ^5^, ^6^, ^7^, ^8^, ^9^ ^]^ As a result, transitioning to an alternate basis of sustainable, renewable, and clean energy is critical for attaining long‐term global security and energy sustainability.^[^ ^10^, ^11^ ^]^ These technologies provide ecologically gracious energy solutions that have the potential to modernize power consumption in recent civilizations. Hydrogen, as an adaptable and essential energy carrier, is critical to the development of sustainable energy resources.^[^ ^12^, ^13^, ^14^, ^15^, ^16^ ^]^ Contrasting batteries, hydrogen has a better storage capacity, which facilitates to mitigate the irregular phase of reproducible energy resources. Hydrogen may be stored in a variety of forms, including condensed liquid, gas, and chemical compounds, which allows it to stabilize the irregular nature of renewable sources such as wind and solar. This feature allows for a predictable and stable energy delivery by turning excess sustainable electricity into the stored hydrogen and utilizing it when sustainable output is less, significantly beyond the storage capacities of present battery expertise. The implications of hydrogen extend across a wide series of characteristics, including fuel cells for the production of electricity, transportation, and heating systems.^[^ ^17^, ^18^ ^]^ For example, electric vehicles powered by hydrogen fuel cells provide a cleaner alternative to regular automobiles.^[^ ^19^, ^20^ ^]^ Hydrogen is also used as a stabilizing agent in industrialized operations, allowing for more environmentally friendly production procedures. Furthermore, hydrogen can be utilized to store excess renewable energy output and released at high demand, allowing renewable energy sources to be integrated into the existing system while preserving stability. The ability of hydrogen to attach to divisions that are complicated to openly electrify, like long‐distance transportation and big industries, emphasizes the necessity of hydrogen in renewable energy sources. By electrolyzing excess sustainable energy, communities can obtain a downy transition to the energy systems while maintaining demand and supply.^[^ ^21^, ^22^ ^]^ To chart a road for an additional ecologically and sustainably friendly prospect, it is necessary to understand the multi‐dimensional role of hydrogen in the greater context of renewable energy. The current energy business faces numerous challenges that jeopardize worldwide energy safety and ecological sustainability. One of the most important challenges is the increased need for energy, which is being driven by rapid industrialization and population growth in growing countries.^[^ ^23^, ^24^ ^]^ Consistent with Benoit,^[^ ^25^ ^]^ growing countries face tremendous challenges in determining the optimum way to use and get more energy to achieve long‐term economic growth. The energy use and high demand for confined expenditure are the primary causes for the requirement to expand the supply of energy in this scenario. The dependence on energy‐intensive procedures is a substantial contributor to the global climate and carbon emission catastrophe.^[^ ^26^ ^]^ Furthermore, reliance on fossil fuels has contributed to Europe's latest energy crises, which have been fueled by economic sanctions, the war in Ukraine–Russia, and growing concern about energy safety inside the EU.^[^ ^27^ ^]^ This expansion requires enormous strain on the infrastructure of present energy, which is primarily reliant on fossil fuels.^[^ ^28^, ^29^, ^30^, ^31^ ^]^ Hydrogen is abundant, produced from sustainable resources, produces zero pollutants, has a high energy content, and provides enduring energy storage, making it an eco‐friendly and feasible alternative to fossil fuels.^[^ ^32^, ^33^ ^]^ The reliance on fossil fuels aggravates weather change by rising emissions of greenhouse gases, which contribute considerably to environmental and global warming concerns.^[^ ^27^, ^34^, ^35^, ^36^, ^37^, ^38^ ^]^ This phenomenon also causes worldwide upheaval, including economic instability and resource wars. In accordance with Kataray et al. and Oyekale et al., integrating renewable energy systems effectively into the grid is another key difficulty.^[^ ^39^, ^40^ ^]^ However, sources like solar, hydroelectric power and wind are renewable; their irregular nature needs vigorous energy generation systems to maintain a consistent energy supply.^[^ ^41^, ^42^ ^]^ Recent generation systems continue to be short of scalability and efficiency necessary to fulfill current society's expectations,^[^ ^43^ ^]^ emphasizing the insistent requirement for renewable hydrogen generation technologies. In consideration of these issues, establishing a renewable hydrogen energy method is critical. Hydrogen provides novel answers to the issues of the energy production industry through proficient emission‐free combustion and energy production.^[^ ^44^, ^45^ ^]^ Using hydrogen as a clean energy source can help to promote cutting carbon emissions, mitigate climate change, and achieve energy independence. Renewable hydrogen systems can mention the concern of irregular sustainable energy systems by excess energy storage as hydrogen during electrolysis and utilizing it through low‐renewable output or high‐demand periods. This ability has the potential to greatly affect inclusive sustainability by electrifying difficult‐to‐decarbonizes sectors such as long‐distance and heavy industrial transportation.^[^ ^46^, ^47^, ^48^ ^]^ The current issues facing the energy industry underline the need for the development and research of hydrogen energy systems. In accordance with Catumba et al., in spite of the raw materials abundance and conventional production methods, investigators are still concerned about the lower yield of hydrogen for a few raw materials, the effectiveness of generation methods,^[^ ^49^ ^]^ the requirement to enhance present techniques, and, most importantly, the challenges linked with leveling up specific raw materials and techniques for significant production of hydrogen. **Figure** **1** illustrates the amount of the global energy supply. Coal accounts for 35% of the global energy supply, followed by gas (23%), hydropower (16%), and nuclear (10%). Sustainable energy sources account for ≈13%, with 7% coming from wind energy, ≈2% from hydrogen, 4% from solar, and the remainder comes from biogas and biomass.^[^ ^50^ ^]^ **Figure** **2** depicts the steps of the hydrogen industry, with a focus on hydrogen generation. The increasing demand for clean energy solutions has propelled interest in sustainable hydrogen production. This review aims to explore various methods and types of hydrogen production, highlighting their applications in different sectors. Furthermore, it will address the challenges faced in the implementation of these technologies and discuss future perspectives for advancing sustainable hydrogen as a key player in the transition to a low‐carbon economy. New catalysts, better electrolysis techniques, and the integration of hydrogen systems with sustainable energy sources are all key fields. This paper seeks to illuminate the potential of hydrogen as a transformative energy carrier by carefully examining these achievements. This also assesses possible options for incorporating hydrogen into existing energy infrastructures, corresponding ecological benefits against profitable implications. This makes sure of an accurate evaluation for future directions of hydrogen expertise. This review explores current industry trends and research achievements to provide approaches for the future possibilities of renewable hydrogen energy sources and offer a critical, in‐depth analysis of the potential and difficulties associated with offshore green hydrogen production using biomass conversion, wind energy, and production by solar energy using a water‐splitting method. Furthermore, it significantly assesses the barriers and constraints impeding the widespread use of hydrogen technology, providing valuable insights to researchers and industry experts on how to approach and overcome these obstacles.

## 2 Methodology

**Figure 3:**
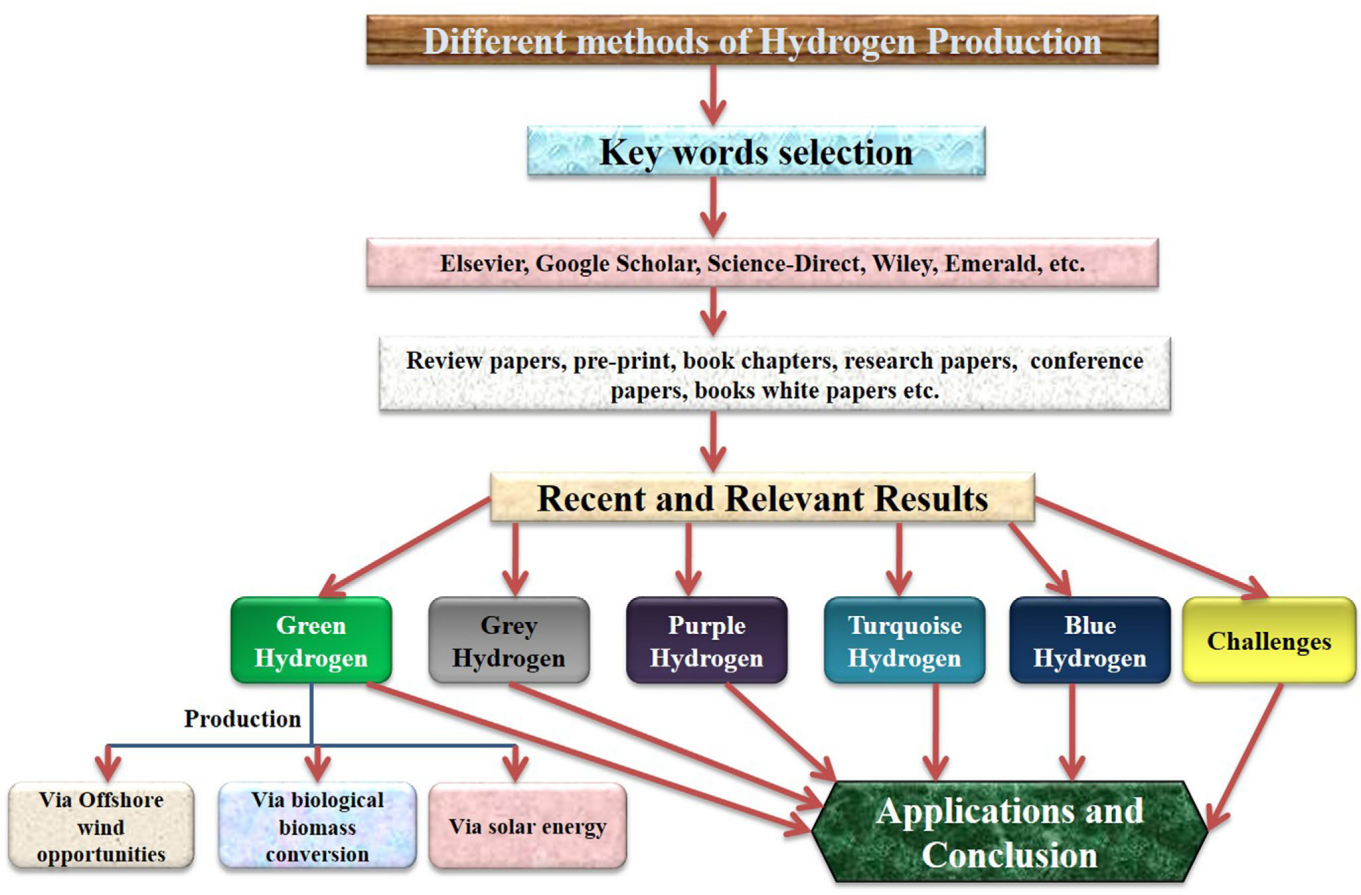
Schematic technique for review.

The examined literature was selected using certain keywords. A detailed technique was employed to examine and gather the most relevant and reputed papers for the evaluation. This is because of comprehensive literature study puts the basis for theory and knowledge development. A comprehensive literature review was undertaken on content delivery. **Figure** **3** depicts the process of conducting five major hydrogen production searches, which comprised purple hydrogen, blue hydrogen, green hydrogen, grey hydrogen, and turquoise hydrogen. Further, green hydrogen production by various processes was also explored. Within the first phase of the collection information process, unpublished materials, abstracts, and research papers were obtained from Elsevier, Science direct, Springer, IEEE, Inder Science, Emerald, Google Scholar, Wiley, and Taylor Francis. Searching for terms like “hydrogen generation”, “hydrogen production”, “hydrogen from biomass”, and “hydrogen from sustainable energy sources” yielded a significant amount of information. The second stage was aimed to extract the most relevant information from recognized sources. Only suitable substances were chosen for further evaluation at the end of the second round. Peer‐reviewed articles and academic papers were taken into consideration. Unpublished manuscripts and abstracts were not considered. The year of publication was used to minimize the numeral, and the bulk of the literature reviewed was published within the recent three years. As they had the latest data, mainly the latest papers were preferred for the study. Additional criteria for selecting the top publications for the study were the methodology, content, and acceptable keywords. Meta‐analyses and systematic reviews were suggested since they provided a comprehensive examination of the reviews and additional knowledge on the study topic. The third stage involves reviewing and categorizing the data based on the publication type, publication year, and characteristics of various sections of the article.

## 3 Hydrogen Production

Production of hydrogen can be divided into three categories: blue (based on natural gas and coal gasification, combined with the CCS Carbon (capture and storage) system of hydrogen generation, grey (based solely on fossil fuels), and green (based on sustainable energy). Green hydrogen can additionally be generated using sustainable energy sources, in spite of the fact that the bulk of its presently formed through the CO_2_‐ methane intensive steam reforming process. Electrolysis is a common process for separating water into hydrogen and oxygen, producing green hydrogen without emitting any carbon dioxide. Sustainable energy resources can be employed to generate the required electrical energy. The cost of manufacturing hydrogen, mainly green hydrogen, is a substantial barrier. The producing cost of one unit of hydrogen via mist reforming is ≈3 times that of 1 unit of energy generated by natural gas. Hydrogen will charge nearly double as much to manufacture by electrolysis at 5 cents kWh^−1^ of electricity as hydrogen generated by natural gas. Low concentrations of hydrogen can be transferred by the existing pipeline infrastructure of natural gas, and it will help to reduce CO_2_ emissions from the current reforming plants of natural gas. Also, a comprehensive cost analysis of hydrogen production can be expressed in terms of either Nm^3^ or kg, providing a clearer understanding of the economic viability of various production methods. For instance, electrolysis, one of the prominent methods, may yield costs ranging from £4 to £7 per kg of hydrogen, depending on electricity prices and system efficiency. Conversely, steam methane reforming typically presents a lower cost of ≈£1 to £3 per kg, but its environmental impact must also be considered. By evaluating these costs in both Nm^3^ and kg units, stakeholders can make informed decisions regarding the most suitable hydrogen production technology for their specific needs.

There are a variety of elements that attract interest to the hydrogen fuel; however, the subsequent are the mainly significant: Various energy resources can be utilized to generate hydrogen. Because hydrogen emits the slightest pollution, it is utilized in combustion or fuel cell processes to produce water. Hydrogen can meet all energy needs and is also used in housing applications, energy carriers, hydrogen stimulated cell vehicles, and incorporated power and heating sources. Hydrogen also meets all energy needs. Hydrogen has the potential to greatly contribute to the marine sector's decarburization by generating clean fuel (ammonia/hydrogen) from offshore off‐grid wind. For instance, catalysts such as platinum and palladium are widely employed in electrolysis and steam reforming processes. These precious metals significantly enhance reaction rates, although their high cost and scarcity pose challenges. Furthermore, emerging materials like transition metal dichalcogenides and nickel‐based alloys are being investigated for their potential to reduce reliance on expensive catalysts while maintaining high activity levels. Additionally, the choice of membranes in electrolysis, particularly proton exchange membranes made from perfluorosulfonic acid, affects both the conductivity and overall efficiency of the hydrogen generation process. Exploring alternative materials and innovations in nanotechnology could lead to more cost‐effective and environmentally friendly methods for hydrogen production. Hydrogen, the ideal substance for this purpose, can transfer and potentially store energy. Various significant hydrogen generation methods include coal gasification, reformation of dry landfill gas, H_2_S methane reformation, natural/methane gas pyrolysis, naphtha reformation, steam reforming of waste oil, the steam‐iron process, the partial oxidation of coal and heavy oil, high‐temperature water electrolysis, grid electrolysis of water, chloralkali electrolysis, PV and solar water electrolysis, biomass gasification, and photolysis of water. According to the research reports and literature,^[^ ^51^, ^52^ ^]^ there are three major types of hydrogen productions based on diverse technologies and potential sources. The color concept evolved due to the employment of key resources in the production of hydrogen. The hydrogen generation from fossil fuels emits CO_2_ and other gases (greenhouse gases). The name “grey hydrogen” refers to this form of hydrogen production and its source.^[^ ^53^ ^]^ Blue hydrogen was created due to combining carbon capture and grey hydrogen technologies to reduce greenhouse gas emissions.^[^ ^54^, ^55^ ^]^ To manufacture hydrogen for sustainable transportation, fossil fuels such as byproduct gas, natural gas, and industrial gas often emit pollutants and greenhouse gases into the environment.^[^ ^56^, ^57^, ^58^ ^]^ A number of technological improvements for hydrogen generation have been described in the literature as a way to reduce pollutants discharged into the environment. As a result, current research^[^ ^59^, ^60^, ^61^ ^]^ has focused on the sustainable energy resources used for hydrogen generation. Green hydrogen is an additional byproduct of electrolysis powered by sustainable energy resources. It has been observed that bioenergy resources like the combustion of biomethane and biomass can produce green hydrogen. Investigators and industries are increasingly focused on improving green hydrogen generation, as green hydrogen generated through a variety of methods has nil gas emissions.^[^ ^62^, ^63^ ^]^

### 3.1 Blue Hydrogen Fabrication Methods

**Figure 4:**
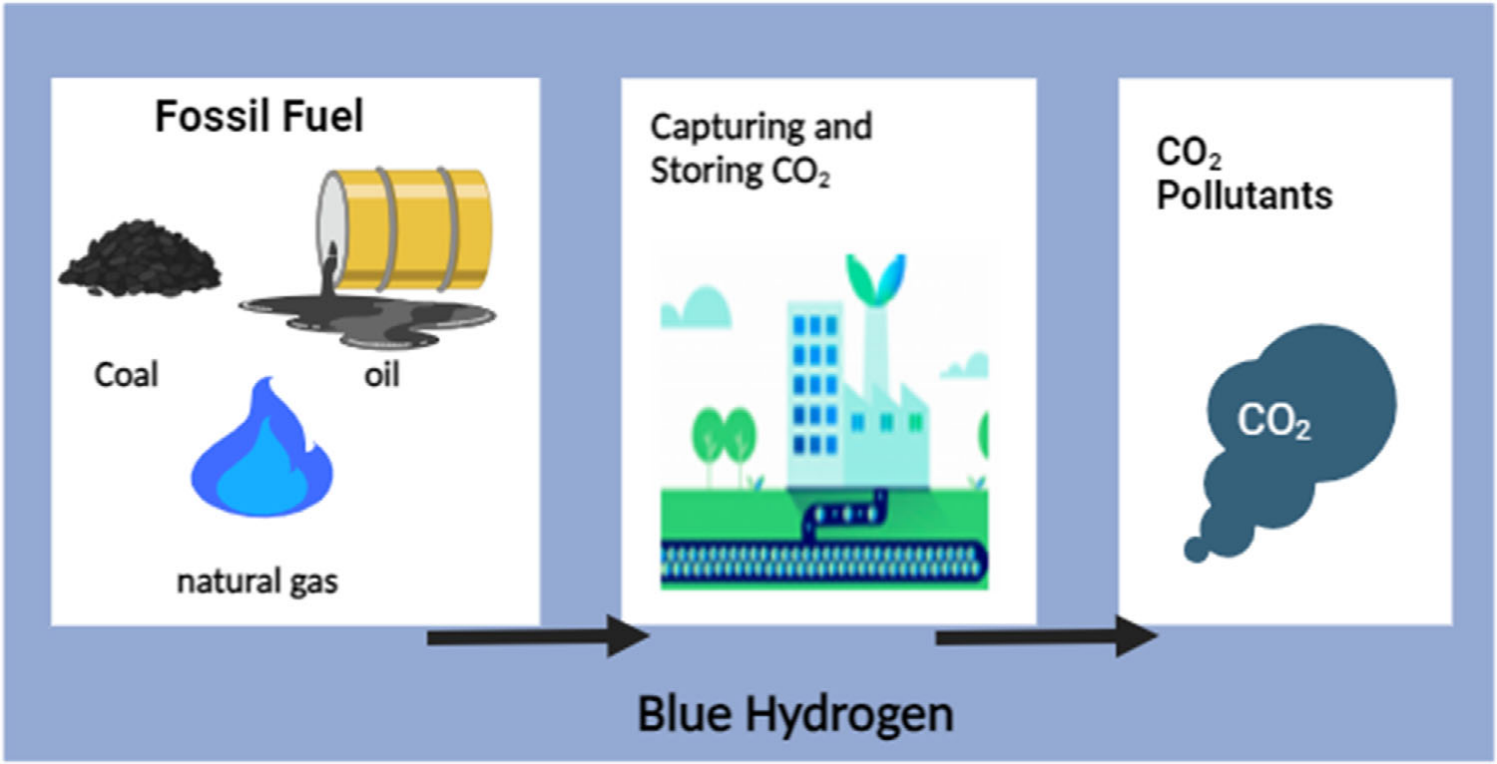
Schematic of blue hydrogen generation (Reproduced with permission from ref. [113] Copyright 2023 MDPI.

Recent usual hydrogen generation techniques use carbon capture utilization (CCU) and carbon capture storage (CCS) technologies to collect CO_2_ discharged emissions. Alternatively, conventional hydrogen production paths are used to meet the world's hydrogen demand, which is now met by sustainable energy sources. These technologies provide a significant amount of hydrogen. The bulk of conservative hydrogen is formed using fossil fuels, primarily by reforming natural gas and moderately oxidizing methane. Natural gas encloses gaseous hydrogen sulfide, which is removed through the first stage of dispensation via desulfurization. The Claus technique is used to separate sulfur from gaseous hydrogen sulfide. Claus plants produce gaseous hydrogen sulfide, which combines with oxygen gas to remove sulfur. Coal gasification is another prominent method for creating hydrogen. Air separation is the first process, in which oxygen is extracted from the air and delivered to the gasifiers. Commonly utilized techniques for air separation are pressure swing adsorption, cryogenic air separation, and membrane separation. Air separation precedes coal pyrolysis. To gasify the coal, the pyrolysis phase requires two inputs: oxygen and steam. This process converts coal into char and volatiles. After the gasification stage, the quenching phase takes place, which involves rapid cooling.^[^ ^64^ ^]^ The next stage is to install a cooling unit for the syngas, which collects heat and can be used for a variety of purposes such as power generation, space heating, and hot water production. The shift in the water gas reaction occurs following the cooling unit of syngas, which transforms CO into CO_2_. Proceeding to the water gas shift, the component separates carbon dioxide (CO_2_) and hydrogen sulfide (H_2_S). Following the hydrogen distillation unit, the removal of an acid gas occurs in which hydrogen is repeatedly separated from the other gases via the swing adsorption pressure technique. **Figure** **4** depicts the procedure for producing blue hydrogen.

### 3.2 Purple Hydrogen Production Method

Purple hydrogen is generated by utilizing nuclear energy, and other types of hydrogen can be made using thermo‐chemical procedures due to the nuclear reactor's high temperatures. The uranium fission generates nuclear energy. The nuclear energy heat is converted into vapor, which is further utilized in turbines to generate electricity.^[^ ^65^, ^66^ ^]^ Power plants don't burn fuel directly; hence, no emission of greenhouse gases takes place. A fission reaction is used because it is controllable in nuclear power reactors. A nuclear power plant's thermal energy is generated by the fission process in the reactor.^[^ ^67^, ^68^, ^69^, ^70^, ^71^ ^]^ Heat generated from the nuclear reactor is utilized to produce steam, which is further used in turbines to generate electricity via a generator similar to those found in conventional thermal power plants. Nuclear energy is generated by the splitting process of a nuclear reactor's atom, which converts water into vapor and runs a turbine to produce electrical energy. The common technique to utilize thermal energy is to spin a turbine for the production of electricity, which can further be used in power cells to produce hydrogen.^[^ ^72^, ^73^ ^]^ Water electrolysis for hydrogen production, thermo‐chemical water splitting phase, and steam reforming processes are some of the other applications for thermal energy.

### 3.3 Turquoise Hydrogen Production

The splitting of hydrocarbons produced turquoise hydrogen. This can be performed through various processes. The plasma technique for creating hydrogen and carbon black is the most advanced research approach and is mostly commercialized. Other methods include methane catalytic conversion, molten metal pyrolysis, and cold plasma through thermal splitting.^[^ ^74^, ^75^ ^]^ Hydrocarbons, like natural gas, are used as both process energy and feedstock in all of these procedures, with electricity serving as the primary source. Methane splitting needs 38 kJ mol^−1^ of hydrogen, whereas water electrolysis needs 285 kJ mol^−1^ of hydrogen, and methane‐steam reforming needs 252 kJ mol^−1^ of hydrogen. Methane splitting necessitates heat losses and extreme temperatures.^[^ ^76^, ^77^ ^]^

### 3.4 Grey Hydrogen Production

**Figure 5:**
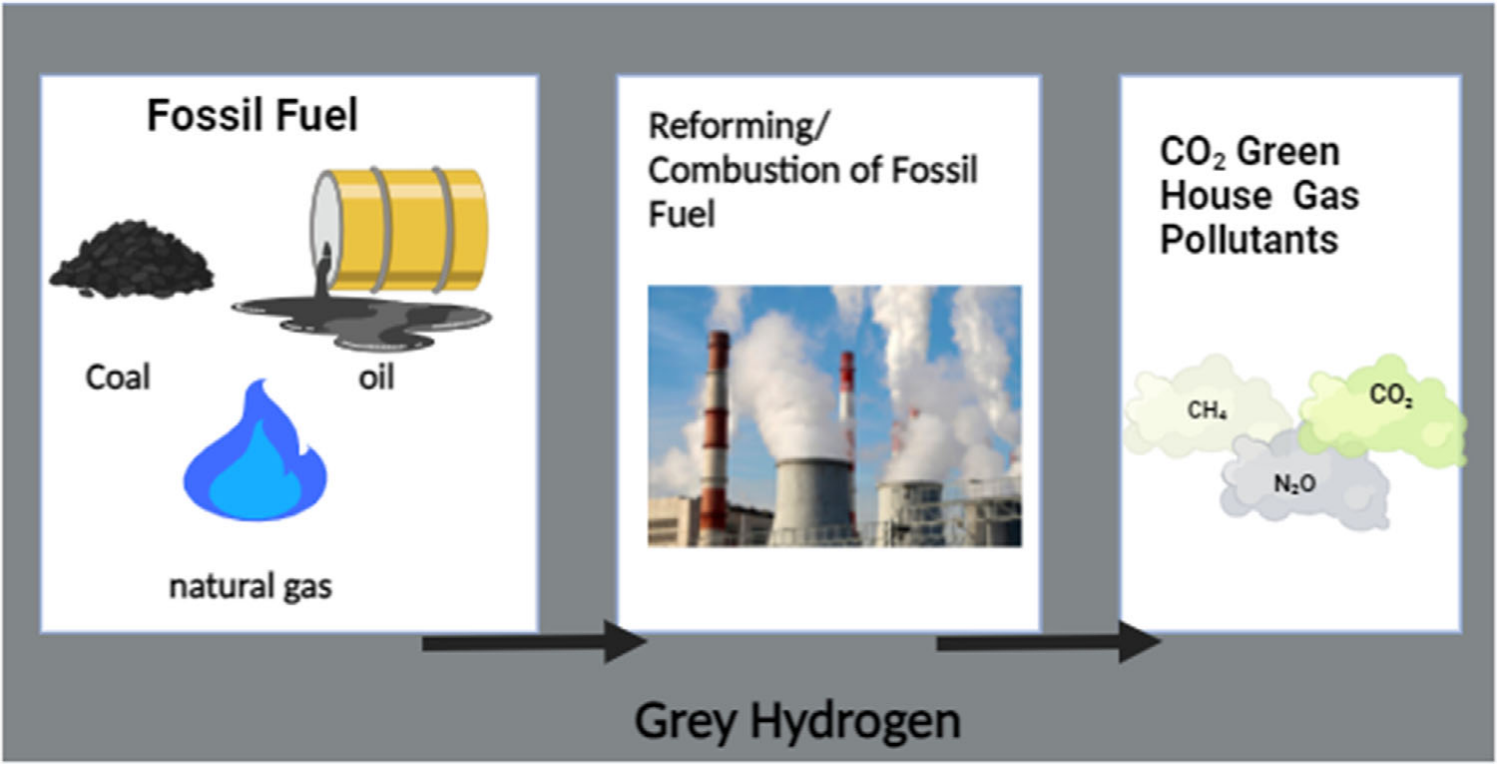
Diagram of grey hydrogen generation (Reproduced with permission from ref. [113] Copyright 2023 MDPI.

Grey hydrogen is the outcome of vapor reforming natural gas or coal with no storage, use, or accumulation of carbon. Over 40% of grey hydrogen is formed as a derivative of other chemical reactions.^[^ ^78^ ^]^ The Freight Efficiency of North American Council has also used the unofficial term “white hydrogen”.^[^ ^79^ ^]^ Grey hydrogen is generally employed in petro‐chemical manufacturing for the manufacture of ammonia.^[^ ^80^ ^]^ The biggest shortcoming of grey hydrogen is the substantial CO_2_ emissions produced through hydrogen production, which are predicted to be ≈830 Mt of CO_2_ per year.^[^ ^81^ ^]^ Steam reforming of natural gas without CCUS is a true and tried approach for producing hydrogen at a reasonable cost. Throughout the method, the water is treated, and natural gas is pretreated. In the reformers, by utilizing steam, methane is converted in syngas. Another notable way of manufacturing hydrogen grey is the gasification of coal, in the literature, it is sometimes known as brown hydrogen. Coal has the world's greatest reserves of several fossil fuels; hence, this technique of production is also widely used. China, particularly, creates a significant amount of hydrogen by gasification of coal due to the coal reserve's abundance and the high cost of natural gases.^[^ ^82^ ^]^ There are four primary forms of coal: sub‐bituminous coal, middle rank bituminous coal, anthracites, and lignite (low rank), which are commonly employed as high‐rank gasification feedstock.^[^ ^83^, ^84^, ^85^, ^86^ ^]^ **Figure** **5** illustrates grey hydrogen production.

### 3.5 Green Hydrogen

**Figure 6:**
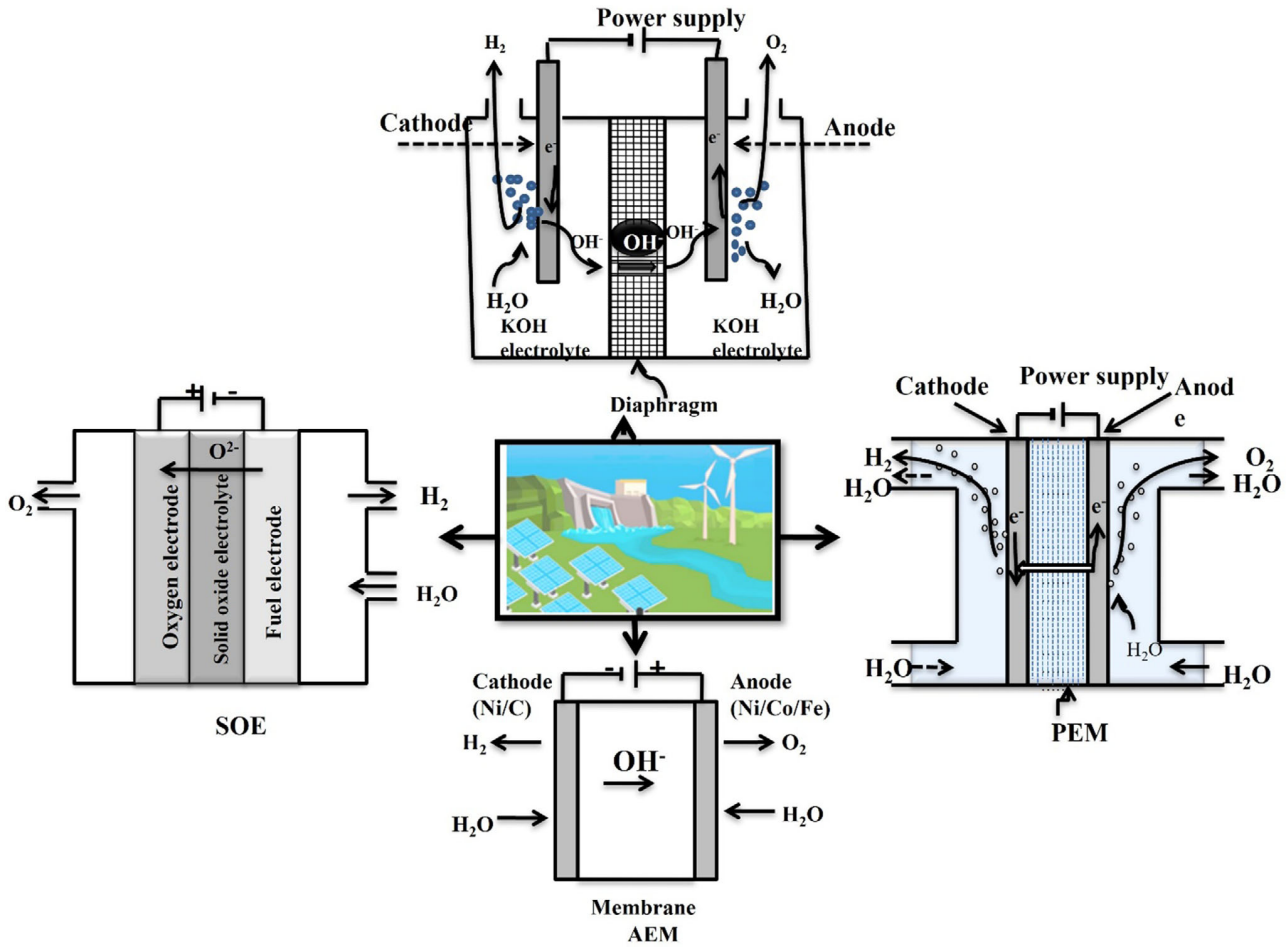
Water electrolysis methods to create hydrogen (Reproduced with permission from ref. [96] Copyright 2024 Elsevier.

The green hydrogen term is used to illustrate hydrogen generation using renewable energy. This (green) hydrogen is formed when water is electrolyzed using the generation of electricity by low‐carbon or renewable power sources. The electrolysis technique is systematically covered before delving into the intricacies of diverse green hydrogen genes. Presently, electrolysis is the mainly well‐established commercially available method of creating hydrogen from water. The electrolysis of water is the method of water dissolving in its constituent components, like oxygen and hydrogen, by employing electric power. The electric potential attracts OH^‐^ to the anode and positive ions (H^+^) to the cathode. **Figure** **6** ^[^ ^87^ ^]^ depicts various water electrolysis methods, including proton exchange membrane (PEM), alkaline water electrolysis (AEL), alkaline anion exchange membrane (AEM), and solid oxide (SOE) water electrolysis. By AEM water electrolysis, predictable noble metal electro‐catalysts could be replaced with less expensive transition metals. Despite being a fairly new technology, AEM electrolysis has sparked interest due to its membrane stability, higher power efficiency, durability, low‐cost hydrogen generation, and simplicity of handling.^[^ ^88^ ^]^ Also, the rise in voltage electrolysis caused by the bubbles formed through the electrolysis practice, excessive energy utilization is an obstruction for the hydrogen production from water.^[^ ^89^ ^]^ Hydrocarbons can be used to reduce energy consumption in water electrolysis. Future electrodes are predicted to be made of low‐cost nonmetal or metal composite materials like nickel. The main sustainable energy resources utilized in the electrolysis practice are: It is widely acknowledged that solar energy is the major sustainable energy resource that can be utilized to generate clean and sustainable hydrogen. Solar energy sources have the ability to play a major role in the alteration to renewable sources from fossil fuels. Solar energy is one of the essential energy resources that could be used to produce clean hydrogen.

**Figure 7:**
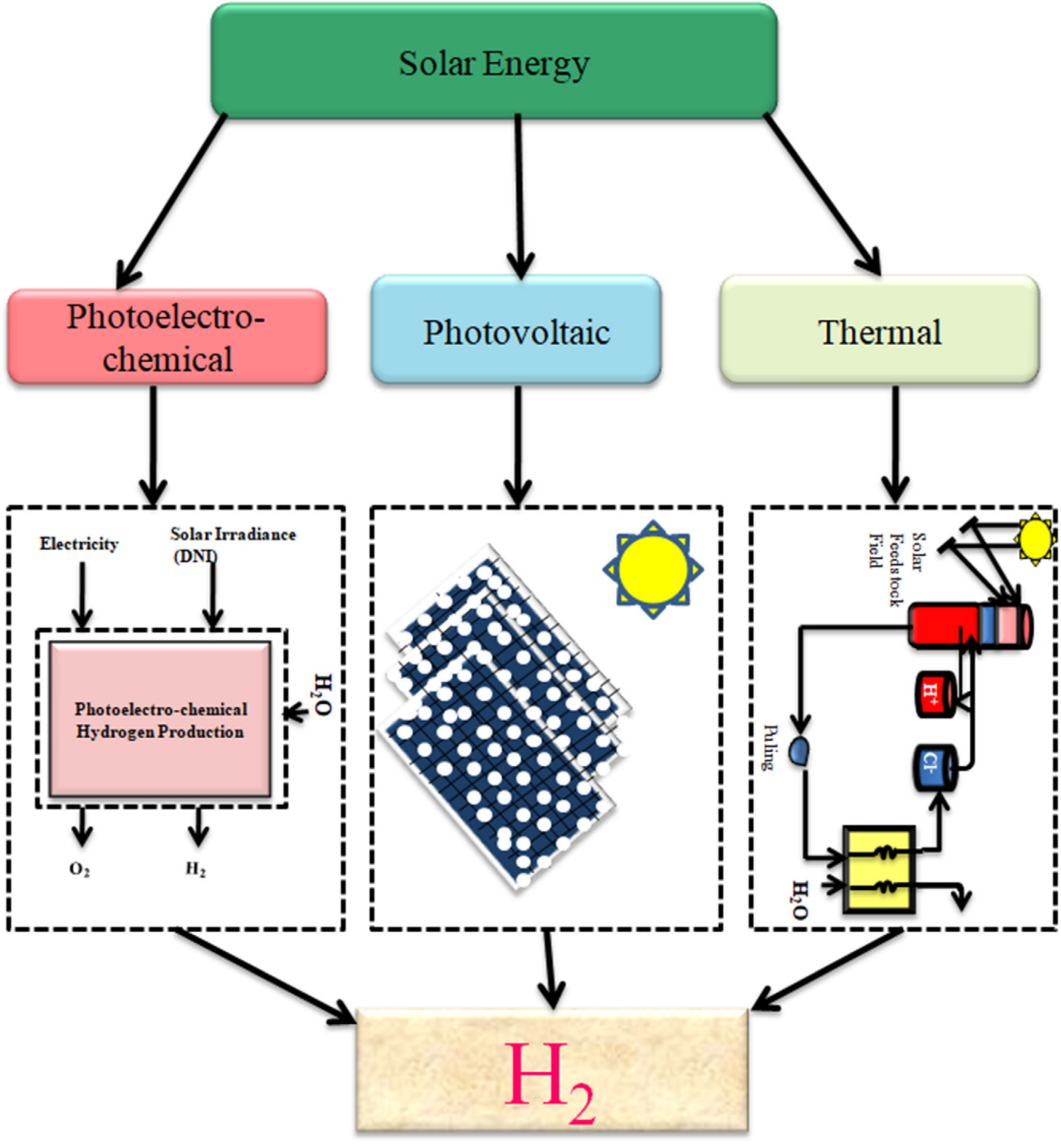
Categorization of solar energy used to generate hydrogen.

**Figure** **7** categorizes the various solar energy resources that can be used to generate hydrogen. The three basic types of solar energy are photovoltaic, photo electrochemical, and solar thermal.^[^ ^90^ ^]^ Hydrogen can be created using the concentrated thermal energy of the sun in various ways, including the solar thermochemical cycle, solar thermolysis, conversion of mechanical energy to electrical energy, solar cracking, electrolysis, and solar gasification. Direct hydrogen production is achievable using both bio‐photolysis and photoelectrolysis. The electricity provided by the photovoltaic resource is used to produce hydrogen during electrolysis. Sunammonia and solar gasification are forming technologies that use rigorous solar energy to manufacture hydrogen. The electrolyzer, which splits water into hydrogen and oxygen, employs the electrical energy provided by the wind energy source once it has been transformed from alternating to direct current.^[^ ^91^, ^92^, ^93^ ^]^ The electrolyzer may use wind power to produce hydrogen, which can then be used in fuel cells to generate energy. Hydrogen can be stored to meet demand during periods of lower wind speed. Using wind power to generate hydrogen is a dependable, cost‐effective, and sustainable source of energy. There are numerous techniques to use geothermal energy to make hydrogen, as well as provided to water electrolysis^[^ ^94^ ^]^ and to heat water to boost the water electrolysis efficiency.^[^ ^95^, ^96^, ^97^ ^]^ Burning biomass in a little amount of air produces carbon dioxide, hydrogen, methane, carbon monoxide, steam, nitrogen, and complexes such as tars, ash, and char particles, all of which include combustible gas.^[^ ^98^ ^]^ Gasification, as disparate to combustion, creates fuel in the syngas form before flaming it, avoiding the emission of several pollutants like nitrogen oxides and sulfur dioxide, and as well as other particles that arise at higher temperatures than those typically used for gasification.^[^ ^99^ ^]^

PEM (Proton Exchange Membrane) water electrolysis offers high efficiency and rapid response times, making it suitable for fluctuating energy sources such as ignocell. However, its reliance on precious metals like platinum for catalysts can lead to higher operational costs. In contrast, ALK (alkaline) water electrolysis is more established and generally less expensive due to its use of non‐precious metals; however, it suffers from lower efficiency and slower reaction rates compared to PEM. AEM (Anion Exchange Membrane) electrolysis presents a middle ground, boasting the potential for lower costs while maintaining a balance between efficiency and operational conditions. Last, SOE (Solid Oxide Electrolysis) operates at high temperatures, allowing for significant energy savings and the utilization of waste heat, but it is typically complex and requires advanced materials to withstand harsh conditions. Each technique presents unique benefits and challenges, influencing their suitability for various applications in hydrogen production.

Additional research results on various methods of hydrogen production are included in **Table** **1**. Because of the great importance of green hydrogen production, we have focused this review on it in further sections.

**Table 1:** Some prominent studies in various types of hydrogen production.

Approach	Significance	Remarks	Refs.
Sustainable and thermodynamic aspects	Solar‐powered hydrogen production	Environmental implications of solar‐powered hydrogen manufacturing	[95]
Thermodynamic aspect	Electrolysis of water on the basis of hydrogen generation	Hydrogen production by the proton exchange on the electrolysis basis.	[96, 97]
Cost assessment carbon emission analysis	Estimation and examination of solar energy power‐driven by natural gas development systems.	Improved energy efficiency and energy	[98, 99, 108]
Thermodynamic modeling	Geothermal energy on the basis of hydrogen generation	Geothermal heat is utilized to warm the water, which is then used in the electrolyzer. It boosts system efficiency.	[109, 110, 111]
Thermodynamic aspect	Hydrogen generation at hydro‐power plants	The efficiency of hydrogen generation improves	[112, 113]
Comparative assessment of renewable energy resources	Production of hydrogen from renewable resources	High‐pressure hydrogen production requires minimal energy consumption.	[114, 115, 116]
Biomass gasification‐on the basis of hydrogen generation	Gasification of biomass on the basis of hydrogen generation	The planned biomass gasification revision demonstrated improved efficiency.	[117, 118, 119, 120]
Energy and energy assessment beside with learning on precise energy utilization	Analysis and assessment of geothermal energy	Geothermal‐powered clean hydrogen liquefaction technique	[121, 123]
Hydrogen infrastructure	future hydrogen infrastructure	This article explores energy security concerns with current fuels and analyses the technological, societal, economic, and infrastructure constraints of implementing new technology.	[124, 125]
Hydrogen production	Future hydrogen promote for industry and production	During the early phase, utilizing long‐term storage, low‐cost, and high‐quality refueling stations yields the best results.	[126, 127]

## 4 Green Hydrogen Production via Offshore Wind Opportunities

Known for its widespread economic feasibility and availability, seawater (near to neutral pH) is viewed as a viable way to energy sustainability when combined with sustainable electricity for hydrogen production. Conversely, pretreatment of seawater to eliminate particles, dissolved salts, and organic matter is required to avoid corrosion, fouling, undesired chemical reactions, membrane and electrode degradation, and salt buildup in the electrolyzer.^[^ ^122^ ^]^ Key issues consist of the generation of hypochlorite and chlorine from the evolution processes of chlorine (Cl_2_) (which occurs in an alkaline medium at ≈1.72 V) that rigorously erodes electrolyte parts. Metal chlorination, ion pollution (e.g., Br^⁻^, SO_4_^2⁻^, F^⁻^, Mg^2⁺^, Na^⁺^, Ca^2⁺^, Sr^2⁺^, K⁺, Cu^2⁺^, HCO^3⁻^, CO_3_^2⁻^, and Cd^2⁺^), and unsolvable Mg/Ca hydroxide's formation on the surface of the electrode can jeopardize the enduring constancy of seawater electrolyzers. Soluble FeCl_3_ dissolved salts can precipitate as Fe(OH)_3_ within the current flow at the cathode, resulting in membrane fouling and electrode. Sediments and microorganisms can primarily cause obstruction of the pore's transport layer and electrode and membrane fouling. To address these challenges, the surface area of the electrocatalytic must be expanded, and the system must be able to efficiently execute both the oxygen evolution reaction (OER) and the hydrogen evolution reaction (HER) during long‐term electrolysis operations of seawater. On the other hand, the separation and purification process should be executed. Several studies have been published thus far on direct seawater electrolysis. It was concluded that OER's (1.23 V) potential for producing hypochlorite/Cl_2_ should be less than that of 1.72 V. The major obstacles of utilizing saltwater for electrolysis are its numerous components, which include bacteria, sediments, and ion species. Furthermore, the composition of saltwater can alter depending on the season and geographic site, affecting its chemical and physical qualities. Direct seawater electrolysis has recently gained popularity worldwide. Yu and his researchers created non‐precious, very stable NiMoN@NiFeN and NiMoN as efficient catalysts for OER and HER, respectively.^[^ ^15^ ^]^ These particles demonstrated constant results at 100 and 500 mA cm^−2^ in a 1 m KOH and seawater solution. Wen and his colleagues used Ir/Ni_1⋅6_Mn_1⋅4_O_4_ as an OER catalyst in an extensive setup with natural seawater and a 6 m KOH solution.^[^ ^100^ ^]^ This mechanism obtained an outstanding 500 mA cm^−2^ result at 1.64 V potential and 60 °C temperature, beating the conventional IrO_2_/Pt‐C mixture. Conversely, when seawater is used alone (without an alkaline solution), there have been relatively few studies of successful and stable oxygen evolution electrocatalysts. As a result, there is a rising curiosity in investigating various corrosion‐resistant and inexpensive transition metal catalysts to improve the deliberate OER process, eventually increasing the selectivity and efficiency of seawater electrolysis. Recent advancements in catalyst technology for seawater electrolysis have focused on developing more efficient and durable materials. Innovations include the use of non‐precious metal catalysts, such as nickel and cobalt‐based compounds, which have shown promising results in enhancing the oxygen evolution reaction. Additionally, researchers are exploring nanostructured catalysts that increase surface area and improve reaction kinetics, thereby boosting overall efficiency. These breakthroughs may significantly reduce costs and enhance the longevity of electrolysis systems, paving the way for more sustainable hydrogen production.

### 4.1 The Offshore Environment's Challenges

Due to the environmental advantages, OWH (offshore wind‐to‐H_2_) schemes are gaining popularity. OWH systems generate H_2_ using renewable energy rather than coal or natural gas, resulting in fresh air and lower carbon emissions. After comparing to earlier offshore wind power schemes, OWH technologies greatly reduce the volatility pressure caused by the renewable energies’ grid connection and provide a viable substitute for the proficient use of abundant offshore wind energy.^[^ ^101^ ^]^ Additionally, OWH systems can transfer energy using already‐existing natural gas pipes instead of constructing new infrastructure for the transmission of electricity, which would lower overall investment. Compared to high‐voltage DC lines in far offshore regions, expected typical expenditure costs and specific costs for energy delivery via hydrogen pipes were determined to be cheaper. There are numerous challenges to organizing OWH systems. The biggest difficulty is economic. The OWH schemes require operational (OPEX) expenditures and significant capital (CAPEX). CAPEX is ascribed to the electrolysis systems, pipelines, and wind farms depending on their location.^[^ ^102^ ^]^ This is in addition to the poor environmental location, which increases construction expenses. OWH systems require expensive vessels for maintenance procedures, which raises OPEX. Strategies to mitigate high CAPEX and OPEX costs include technological innovations that enhance efficiency, economies of scale through larger projects, and government incentives or subsidies to support initial investments. Additionally, developing robust supply chains and optimizing maintenance processes further reduce overall expenses, making OWH systems more competitive. Long‐term water erosion also reduces the components’ service life compared to onshore systems. Moreover, leading‐edge corrosion in wind farms can be caused by strong gusts. Last, it is necessary to discuss an economic approach to how an OWH network connects into other parts of the supply chain, covering market caps, demand, and so on. In this context, different accessible technologies are compared using the standardized price of energy (LCOE) as a baseline. The system's expenses and electricity output are linked by the LCOE. Moreover, the lowest market price needed to produce a 10% yearly exchange rate during the system's overall duration is determined by the leveled expenditure of H_2_ (LCOH).^[^ ^19^ ^]^ The LCOH over the system's lifetime is expressed as follows in other literature: The next issue concerns the technological aspects of OWH systems. When building the OWH systems, keep in mind the unfavorable and variable offshore environment, which includes wind, seabed, and wave topography.^[^ ^103^ ^]^ These options make the construction procedure and technical specs difficult for either floating or fixed scenarios. Because of the high cost of offshore maintenance, the components must be designed to be run without maintenance for an extended period of time. The created H_2_ can be transmitted onshore via pipes or kept in containers that can then be used up or moved onshore in situ by other maritime vessels; thus, H_2_ demand and supply scenarios must be painstakingly arranged. Next one, there are ecological concerns that must be noticed.^[^ ^104^, ^105^ ^]^ These comprise the impact on the wildlife species both above and below the water. Bergstrom et al. found that underwater systems have positive impacts such as fisheries exclusion and habitat gain, while negative impacts include noise disruptions and electromagnetic fields.

### 4.2 Offshore Platform's Selection

**Figure 8:**
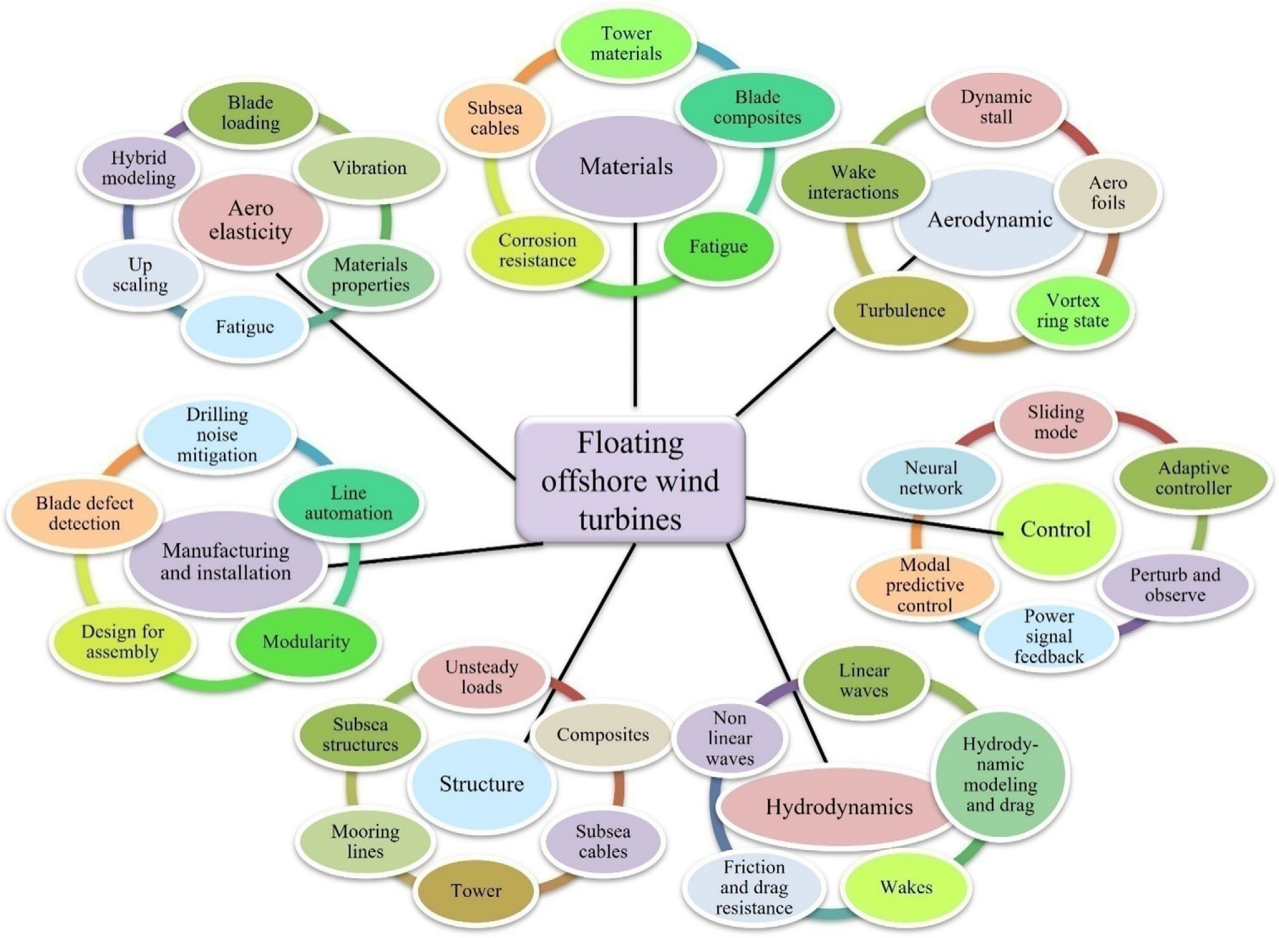
Research thread is significant to floating offshore wind turbines (Reproduced from the open access Journal of Elsevier 2024).^[^ ^15^ ^]^

Choosing an appropriate offshore platform will be determined by the water's state and local winds, seabed, wind turbine size, production facilities, harbor depth, and the availability and cost of equipment and materials. Rezaeiha and Micallef evaluated the existing study on suspended offshore wind turbines (**Figure** **8**).^[^ ^106^ ^]^ The location of the offshore plant has a significant impact on the facility's design. Wind speed, weather conditions, water depth, and wave height must all be considered while determining the ideal position for the capability. The platform must be built to be easily available for repair and maintenance, which demands the installation of appropriate walkways, access points, and other essential infrastructures. Last, all appropriate national, international, and local regulations and laws governing offshore hydrogen and maritime operations must be considered while developing the plant. Corrosion, severe temperatures, and seawater are all part of the hostile marine environment. These days, there are four different kinds of offshore wind turbine platforms: spar, barge, semi‐submersible, and TLP (tension leg platform). Fixed wind turbines are not feasible because over 80% of the globe's offshore wind sources are found in oceans deeper than 60 m. Three coupling scenarios—centralized onshore, decentralized offshore, and centralized offshore electrolysis solutions—were suggested by Ibrahim et al., who focused on large‐scale floating OWH systems. They came to the conclusion that PEM (proton exchange membrane) electrolysis is a more effective solution because of its small size and operational capabilities (rapid dyna), while alkaline electrolysis is advantageous for the centralized onshore (lower CAPEX but longer response time). Finally, for centralized offshore, either PEM electrolysis or alkaline electrolysis can be used; however, since the best choice is heavily reliant on wind conditions, a more thorough decision‐making procedure is required. Three factors were examined: the platform, the energy transfer mechanism, and the electrolysis process. To identify connected hydrogen storage and wind power systems, Wu et al. created a two‐step site decision‐making framework.^[^ ^107^ ^]^ To determine the criteria assessment weight, they employed the fuzzy entropy approach. They discovered that the ideal locations for H_2_and wind project coupling should be determined using the VIKOR and TOPSIS techniques. **Table** **2** represents global efforts and projects on offshore wind to green hydrogen production.

**Table 2:** World‐wide efforts to produce green hydrogen using offshore wind.

Project/company	Year deployed	Capacity/details	Country	Refs.
European clean hydrogen partnership program — the hydrogenoffshore production for Europe (HOPE)	2026	10 MW 4 tons day^−1^ of green hydrogen	Belgium	[15]
OceanREFuel project	2021	Ocean REFuel is a five‐year research project funded by the Physical Sciences and Engineering Research Council (epsrc) with the goal of turning ocean‐renewable energy into gaseous and liquid fuels	UK	[21]
Lhyfe/sealhyfe	2022	1MW to 0.5 tones per day Associated with SEMREV power hub, the first floating wind farm in Europe	France	[34]
Siemens energy and siemens gamesa – H_2_Mare	2021	Electrolysers with 3.5 MW additionally, taking into account the conversion of hydrogen to ammonia, methanol, methane, and liquid hydrocarbons	Germany	[59]
NortH2 — a consortium consisting of RWE, equinor, eneco, and shell	2020	Aiming for 4 GW of offshore green hydrogen by 2030 and 10 GW or more by 2040	Netherlands	[65]
AquaVentus (SUBPROJECTS: AquaPrimus, Aqua Ductus, AN D AquaSector)	2020	10 GW by 2035	Germany	[64]

### 4.3 Green Hydrogen Production via Biological Biomass Conversion

**Figure 9:**
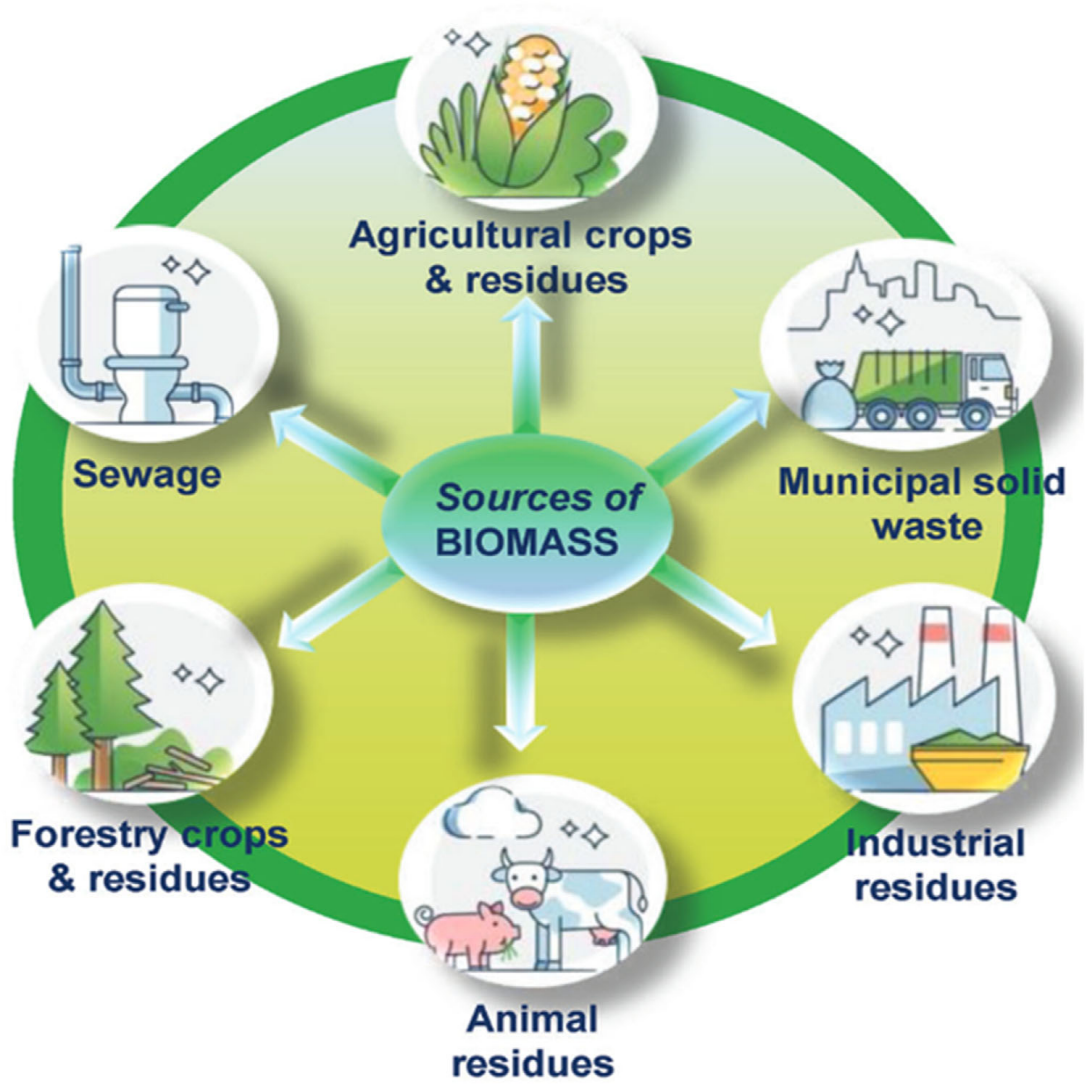
Biomass resources encompass a wide range of feed stocks Reproduced with permission from Ref. [164] Copyright 2023 Wiley.

Hydrogen generation from biomass can be divided into two categories: biological biomass and thermo‐chemical conversion techniques. As previously noted, thermo‐chemical pathways include coal gasification, pyrolysis, steam reforming, partial oxidation, and the thermo‐chemical cycle. Biomass resources are organic materials that are derived from living organisms, and they can be utilized as a source of solar energy and valuable chemicals. Biomass resources encompass a wide range of feedstocks like forestry wastes, agricultural residues, industrial residues, and municipal and sewage solid wastes (**Figure** **9**).

**Figure 10:**
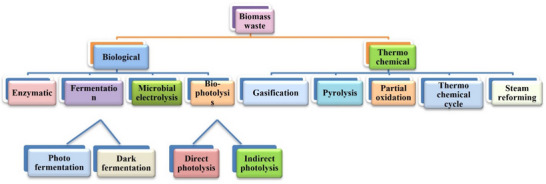
Hydrogen production from organic waste and biomass including thermo‐chemical and biological routes.

Fermentation, biophotolysis, enzymatic, and microbial electrolysis are the four major biological conversion routes.^[^ ^13^ ^]^ Bio‐photolysis and fermentation can be further classified as photo and dark fermentation and indirect and direct bio‐photolysis, respectively (**Figure** **10**). Over 96 percent of the worldwide H_2_ is produced by the thermo‐chemical hydrogen route, which includes 18% of coal gasification, 30% of oil cracking, and 48% natural gas steam reforming. However, just 1% of the world's total H_2_ output comes from the synthesis of hydrogen from biomass. Because of their high hydrogen content, organic solid wastes like cellulose and hemicelluloses are desirable sources of hydrogen. On the other hand, lignin converts to hydrogen at a low rate.^[^ ^33^ ^]^ Carbohydrates have also been found to contribute to the production of hydrogen. Because of its elevated H: C ratio (pure cellulose: C_6_H_10_O_5_ = 1.7 vs 0.8 for fossil fuels), plenty, and ecological viability, biological biomass becomes a more attractive option than fossil fuels from an economic and environmental perspective. Three categories can be used to classify biomass resources. Agricultural waste derived from crops, forests, and tree residues; lignocellulose biomass comprises cellulose (Mol Wt 162.14 g mol, 33–51%), hemicelluloses (Mol Wt 132 g mo1, 19–34%), and lignin (20–30%). In addition to organic components with high moisture, municipal solid waste (MSW) comprises inorganic components such as metals, glass, wood, food leftovers, paper, leather, and plastics. Thus, dehydration and separation are required before using the organic portion of MSW to produce hydrogen. Non‐lignocelluloses biomass is produced by microbes, animals, and a fraction of plants, and it is primarily composed of proteins, carbohydrates, and lipids, similar to macro and microalgae.^[^ ^40^ ^]^ The chemical composition of lingocellulosic biomass from different sources is shown in **Table** **3**. Although, there are several difficulties in converting organic solid waste and biomass to hydrogen, including seasonal accessibility, high treatment, and handling costs, tar and char production, and restrictions due to factors like corrosion, aging, and pressure. Thus, these challenges must be conquered in order to reach better conversion efficiency of hydrogen. It will primarily focus on biophotolysis by cyanobacteria, specific microalgae, and microorganisms. Biophotolysis by microorganisms is the process by which microorganisms divide water molecules (H_2_O) into hydrogen (H_2_) and oxygen (O_2_) molecules in the existence of light amid no carbon emissions. These microbes produce the enzymes, structural components, and pigments required for biophotolysis. Plants absorb sun energy (photons) and create a powerful oxidant that converts molecules of water into O_2_, H^+^, and electrons. Hydrogenase is the enzyme that recombines H^+^ into H_2_ gas. However, it was discovered that O_2_ formed by bio‐photolysis and photosynthesis inhibits the action of hydrogenase.^[^ ^99^ ^]^ Thus, to neglect this problem, the O_2_ content should be kept at or below 0.1%. It can be accomplished by reducing O_2_ generation or using O_2_ binding proteins. Furthermore, improper utilization of solar energy for the synthesis of hydrogen occurs from photosynthetic cells like chlorophyll and other pigments absorbing up to 90% of bright light as heat energy. The electrons or energy required to catabolize molecules produced throughout the photosynthesis reaction in order to generate hydrogen are shown above. There are two steps involved in direct bio‐photolysis. In the first stage, photo systems use algae or cyanobacteria to produce carbohydrates by absorbing CO_2_ and H_2_O in the existence of light. Water splits into electrons and H^+^ when exposed to light, producing O_2_. Both H_2_ and O_2_ are supplied to the fuel cell, which generates energy and water after hydrogenase reassembles H^+^ into H_2_ gas.^[^ ^80^ ^]^ A long‐term cyclic path is ensured by recycling this water back to the photo system. It is also possible to store the produced hydrogen for later use. The two microorganisms that are most frequently studied and utilized for hydrogen generation are microalgae and cyanobacteria.

**Table 3:** The chemical composition of raw biomass.^[^ ^40^ ^]^

Biomass	Cellulose [%]	Lignin [%]	Hemicellulose [%]
Sugarcane bagasse	32–48	23–32	19–24
Sugarcane staw	40–44	22–25	30–32
Hard wood	43–47	16–24	25–35
Corn stover	35	35	25
Soft wood	40–44	25–31	25–29
Corncob	45	15	35
Wheat straw	30	15	50
Cotton	95	0.3	2
Sisal hemp	73.1	11	14.2
Coir	36–43	41–45	0.15–0.25
Rice straw	43.3	16.3	26.4
Banana fiber	60–65	5–10	6–8
Switchgrass	30–50	5–20	10–40
Barley straw	31–45	14–19	27–38
Rye straw	33–35	16–19	27–30
Rice husk	25–35	26–31	18–21
Oat straw	31–37	16–19	27–38
Sweet sorghum	34–45	14–21	18–27
Bamboo	39.8	20.81	19.49
Corn leaves	26.93	15.18	13.27
Hazelnut shell	25.2	42.1	28.2
Herb	38	22	24
Cork	44	30	21
Miscanthus	38–40	24–25	18.24

### 4.4 Green Hydrogen Production via Solar Energy

Sun‐driven hydrogen evolution methods use solar energy to create hydrogen via a variety of processes. Photoelectron chemistry (PEC) and PC water splitting are two typical techniques to solar‐driven hydrogen evolution. Each method has advantages and disadvantages, and the best option is determined by the unique application and requirements.

#### 4.4.1 PEC Water Splitting

Sustainable energy sources are essential due to the imbalance between global population increase, rising energy consumption, and declining fossil fuel supply. The search for renewable and environmentally friendly energy sources has garnered a lot of attention upward to this point. Improving solar energy collecting and storage is necessary due to intermittent sunlight and supply‐demand imbalances.^[^ ^128^ ^]^ PEC H_2_O splitting employing earth‐abundant semiconductor electrodes from the solar energy translation and storage revolt has gotten a lot of interest. If the goal of successfully developing and implementing hydrogen‐based fuel systems is met, it could pave the way for hydrogen to be widely used as an environmentally beneficial fuel. To use a semiconductor electrode for PEC H_2_O splitting, it must meet several criteria, including an appropriate band gap, suitable valence and conduction band places for water oxidation and reduction, effective visible light absorption, chemical stability, and commercial viability.^[^ ^129^, ^130^, ^131^ ^]^ Fujishima and Honda's catalyst research piqued academics’ interest in PEC water splitting in the 1970s. They were able to split water using a PEC cell with a single crystalline TiO_2_ (rutile) anode and a Pt cathode, as well as UV radiation and an external bias. Although PEC water splitting systems have proven potential in a variety of applications and accomplished multiple significant milestones, they continue to confront a number of challenges/limitations, including complex setups, electrode selection, and stability over larger surface areas, and electrode corrosion. This indicates that the quest for H_2_O splitting photoelectrode materials is currently ongoing. Researchers are exploring photocatalytic water splitting to address constraints in PEC water splitting.

#### 4.4.2 Photocatalytic Water Splitting

**Figure 11:**
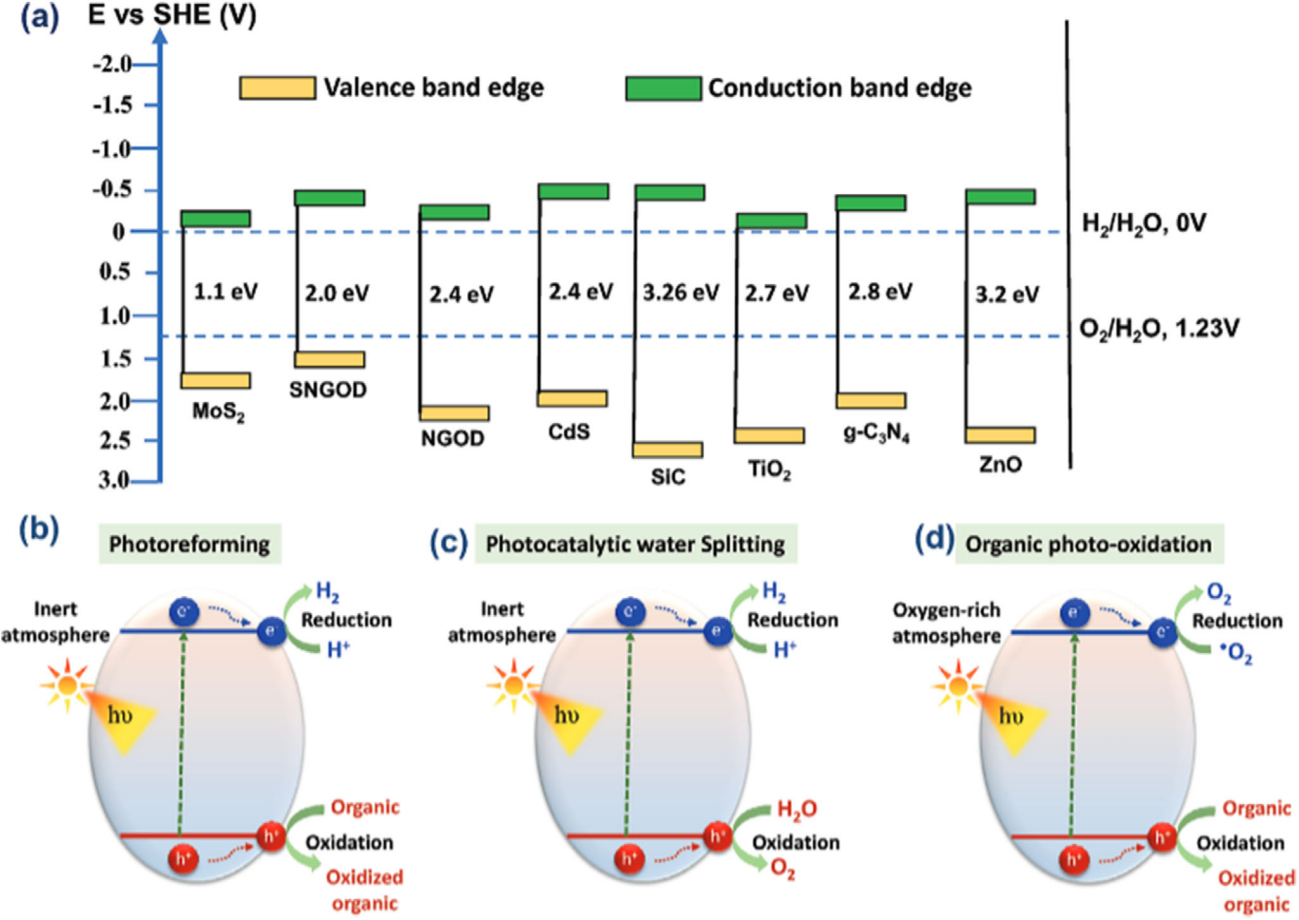
a) Band gap position of some common photocatalysts used for photoreforming, and the pathways of b) photoreforming c) Photocatalytic water splitting in anaerobic conditions and d) Organic photo‐oxidation in the presence of oxygen. Reproduced with permission from ref. [164] Copyright 2023 Wiley.

Following the landmark study undertaken by Honda and Fujishima in the 1970s, which demonstrated the PEC H_2_O splitting into hydrogen and oxygen, several techniques and photocatalysts have been used for catalytic H_2_ synthesis driven by solar light. The most fascinating and challenging of these approaches is photocatalytic H_2_ generation from water using SEDs under ambient circumstances (pressure and temperature) (**Figure** **11**c). This technique is a self‐contained process that captures and converts solar energy into chemical energy. Photocatalytic hydrogen evolution is a promising approach due to its simplicity, flexibility in catalyst selection, stability, and scalability. To achieve the best semiconductor for H_2_O splitting, the VB and CB edge positions should be sandwiched between the potentials for H_2_O oxidation and reduction (i.e., *E*_v_ < *E*_ox,water_ and *E*_c_ > *E*_red,water_). Figure 11a displays the band edge positions of some semiconductors relative to H_2_O redox potential. Various MOFs, ZIFs, metal oxides, and sulfides photoelectrode materials, such as TiO_2_, CdS, MoS_2_, GaP, ZnO, g‐C_3_N_4_, SiC, UiO‐66, ZIF‐8, graphene oxide dots, carbon dots (CDs), and PANI, have since been created for PEC H_2_O splitting.^[^ ^132^, ^133^, ^134^, ^135^, ^136^, ^137^, ^138^, ^139^, ^140^, ^141^ ^]^ The process of oxidizing H_2_O to obtain electrons in a photocatalytic device consumes a significant amount of energy. Most artificial photocatalytic systems for hydrogen production involve sacrificial substances. These compounds act as electron donors, scavengers, and hole carriers to reduce protons, preventing e^−^ and h^+^ recombination and improving the process's efficiency.^[^ ^142^, ^143^, ^144^, ^145^, ^146^ ^]^ The H_2_O splitting reaction is a significant uphill process, with a Gibbs free energy change of Δ*G* = +237.1 kJ mol^−1^. Photocatalysis with a sacrificial donor remains tough, but less difficult than water splitting.^[^ ^147^, ^148^ ^]^ Currently, the majority of H_2_ is produced from fossil fuels such as coal, oil, and natural gas, with biomass accounting for a minor percentage.^[^ ^149^, ^150^ ^]^ Producing hydrogen from sustainable sources such as biomass, water, and solar energy is possible but poses significant obstacles.^[^ ^151^ ^]^ Commonly used sacrificial agents for H_2_ evolution, such as tri‐ethanolamine (TEOA), sulfurides, and methanol, are costly and non‐renewable, creating a downside.^[^ ^152^, ^153^ ^]^ When compared to traditional photo catalytic methods, photo reforming of biomass provides an audacious means of generating sustainable and scalable H_2_.^[^ ^154^ ^]^

## 5 Hydrogen Utilization

### 5.1 Industrial Applications

**Figure 12:**
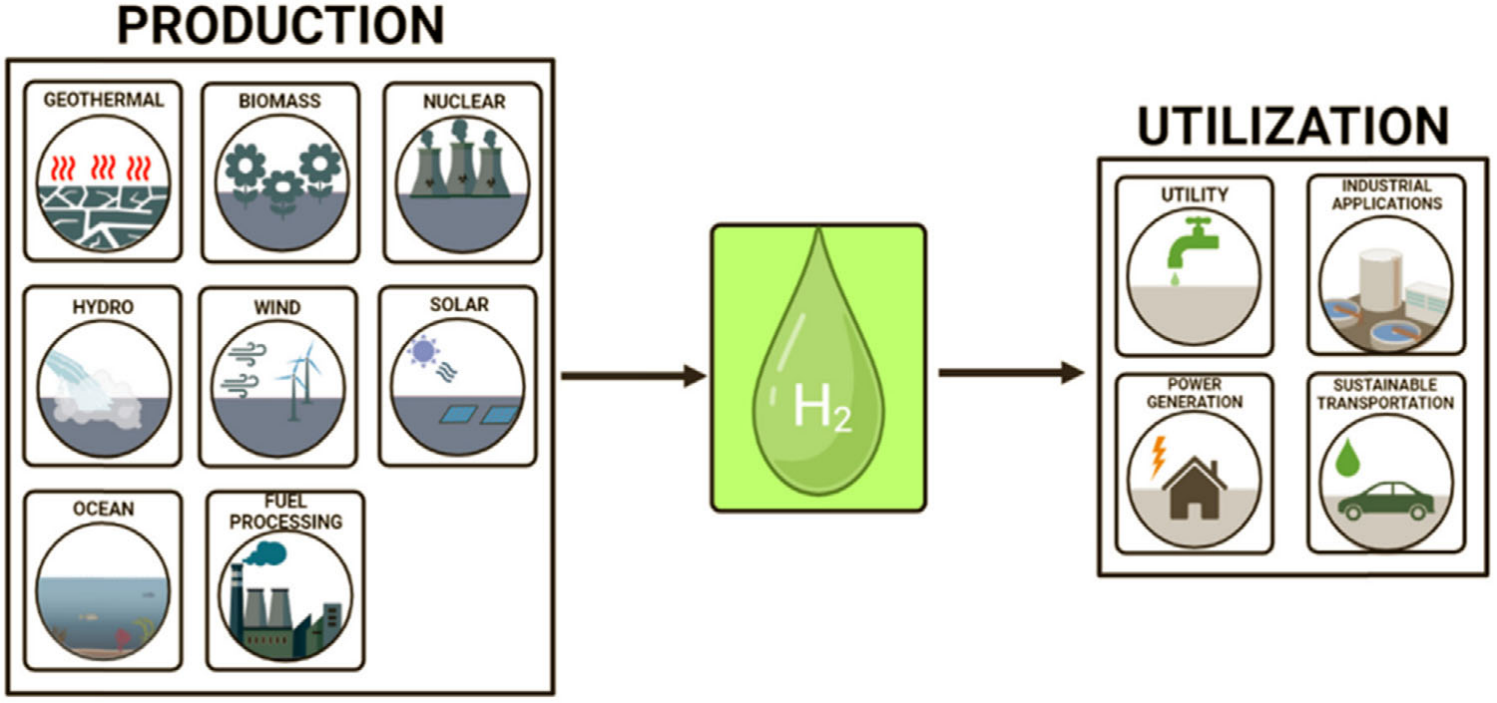
Various elements of hydrogen generation and Reproduced with permission from ref. [162] Copyright 2023 Chemical Society RSC.

The two main uses of hydrogen are as an energy transporter and as an antioxidant in industrial settings. Due to its enormous energy storage and transfer capacity, hydrogen should be viewed as an energy carrier instead of an energy producer. Light is used as an energy source in photo electrochemical cells to split water into oxygen and hydrogen. At the moment, hydrogen is primarily utilized in the refinement of petroleum, the production of fertilizer based on ammonia, and emerging sectors such as utilities and transportation (**Figure** **12**), where it is employed in energy storage, power generation, commercial and residential heating, industry, aviation, road transportation, and shipping. An estimated 70 million tones of hydrogen are generated annually for a range of industrial uses, such as metallurgy, food processing, fertilizer and chemical manufacturing, ammonia production, steel and methanol industries, and oil refining. This hydrogen can be combined with other substances or left pure. Nowadays, ≈3%, 27%, 33%, and 11% of hydrogen is used for steel production, ammonia production, oil refining, and methanol production, correspondingly.^[^ ^155^ ^]^ In addition, hydrogen is used as an oxygen scavenger in corrosion prevention and oxidation applications, as a coolant in cryogenic operations, and as a green fuel in rocket engines and fuel cells. The hydrogen used as an energy transporter to reach net zero carbon emissions and maintain the global typical temperature less than 1.5 °C above pre‐industrial levels by 2050 is predicted to drive a 3–10 times increase in global hydrogen demand (GHD) compared to today (Paris Agreement, 2015). The GHD is anticipated to be 500 Mt by the International Energy Agency and 539 Mt by the Hydrogen Council.^[^ ^155^ ^]^ About 66.5 Mt will be used for power generation, 40.8 Mt for syngas invention, 62.9 Mt for industrial uses, 26.6 Mt for building applications, 16.9 Mt for refining applications,18.3 Mt for ammonia fabrication, and the remaining portion for transportation, according to the total estimated GHD in 2050.^[^ ^156^ ^]^ Hydro cracking, which breaks down large hydrocarbons into smaller ones, and hydro treatment, are the two primary hydrogen‐consuming steps in the oil refinery process. Oil refining uses ≈33% (38 Mt H_2_ annually) of hydrogen. Hydrodesulfurization (which turns S into H_2_S), hydro isomerization (which turns paraffin into isoparaffins), and dearomatization (which turns aromatics into alkanes and cyclo‐paraffins) are a few examples of hydro treatment. While hydrogenation stabilizes certain chemical compounds, hydro cracking breaks down long‐chain hydrocarbons into shorter chains. Desulfurization of hydrocarbons to create fuels free of sulfur is one of the most significant uses of hydrogen. Since sulfur is found naturally in fossil fuels like crude oil, hydrogen reacts with it to create H_2_S (Equations (1),(2)).

Hydro desulfurization

(1)

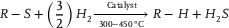




Hydrodenitrogenation

(2)

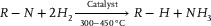




If sulfur is not removed during this process, it can be oxidized to form SOX (SO_2_, SO_3_). Sulfur oxides are environmental contaminants that cause a variety of concerns, with respiratory troubles in kids, as well as smog and acid rain in cities. Sulfur can also interact with and impair catalytic exhaust systems. During desulphurization or softening, organic sulfur interacts with hydrogen at high temperatures (4350 °C) and high pressures (60 bar) to form H_2_S, which, as observed in refineries, can separate from the framework and react with air to form yellow sulfuric compounds.^[^ ^157^, ^158^ ^]^ As a result, this solid yellow product has industrial applications.

### 5.2 Fuel Applications

#### 5.2.1 Aeronautics and Space Sectors

**Figure 13:**
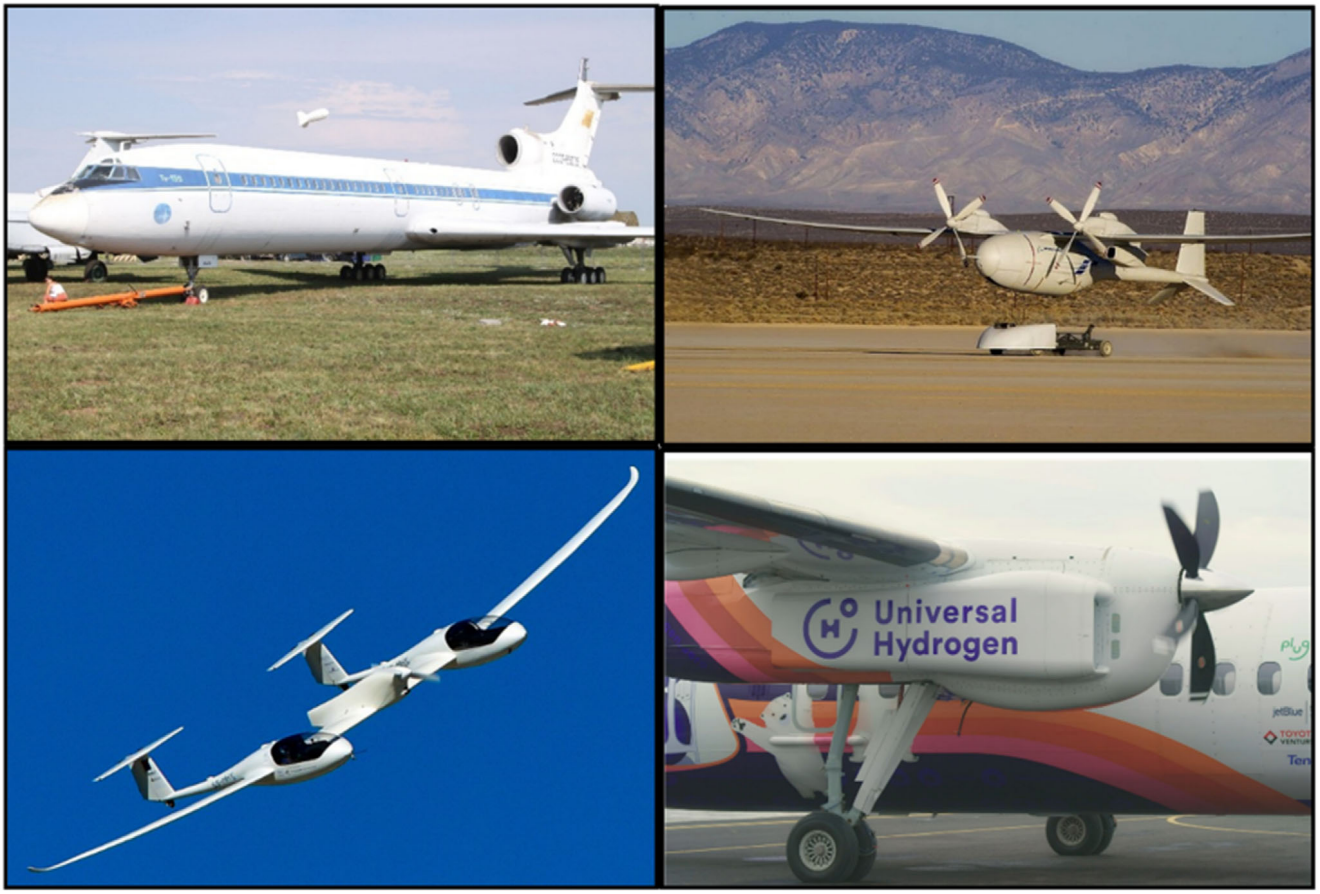
The Tupolev Tu‐155, located in the top left, is a customized variant of the Tupolev Tu‐154 (CCCP‐85035) utilized as a testbed for substitute fuels. Top right: Boeing Phantom Eye UAV powered by hydrogen. Bottom left: HY4 powered by a hydrogen fuel cell. Universal Hydrogen launched the Dash B‐300 turboprop. Reproduced with permission from ref. [162] Copyright 2023 Chemical society RSC.

Fuel cells are crucial for providing control to spacecraft and satellites, especially in the realm of space investigation. During Apollo missions of NASA in the 1970s and 1960s, fuel cells were utilized to supply drinking water and produce electricity for astronauts.^[^ ^159^, ^160^, ^161^, ^162^, ^163^, ^164^, ^165^, ^166^, ^167^, ^168^, ^169^, ^170^, ^171^, ^172^, ^173^ ^]^ These fuel cells utilized hydrogen and oxygen as fuel resources and functioned through an electrochemical mechanism referred to as the fuel cell of PEM. In addition to producing drinking water as a byproduct of the chemical reaction among hydrogen and oxygen, the fuel cells produced electricity for the spacecraft. During the Apollo missions, fuel cells demonstrated efficiency and reliability in the challenging conditions of space, contributing to the safety and success of astronauts on their lunar journey.^[^ ^161^ ^]^ Hydrogen has been utilized in aerospace applications for several decades (**Figure** **13**). The Lockheed Aircraft Corporation, an American aerospace producer, developed a secret CL‐400 prototype aero plane in 1956, which carried 9.74 tons of liquid hydrogen.^[^ ^164^ ^]^ In 1988, the Soviet Union developed the TU‐155 profitable jet, featuring three engines, one of which operated on liquid hydrogen (Figure 13, top left panel).^[^ ^165^ ^]^ This aircraft is recognized as the world's first tentative model utilizing hydrogen as a fuel source. The aircraft was utilized to evaluate the safety, technical feasibility, environmental compatibility, and economic viability of employing liquid hydrogen as fuel. Recent tests demonstrated the successful performance of BE‐3 rocket engines powered by liquid oxygen and liquid hydrogen.

**Figure 14:**
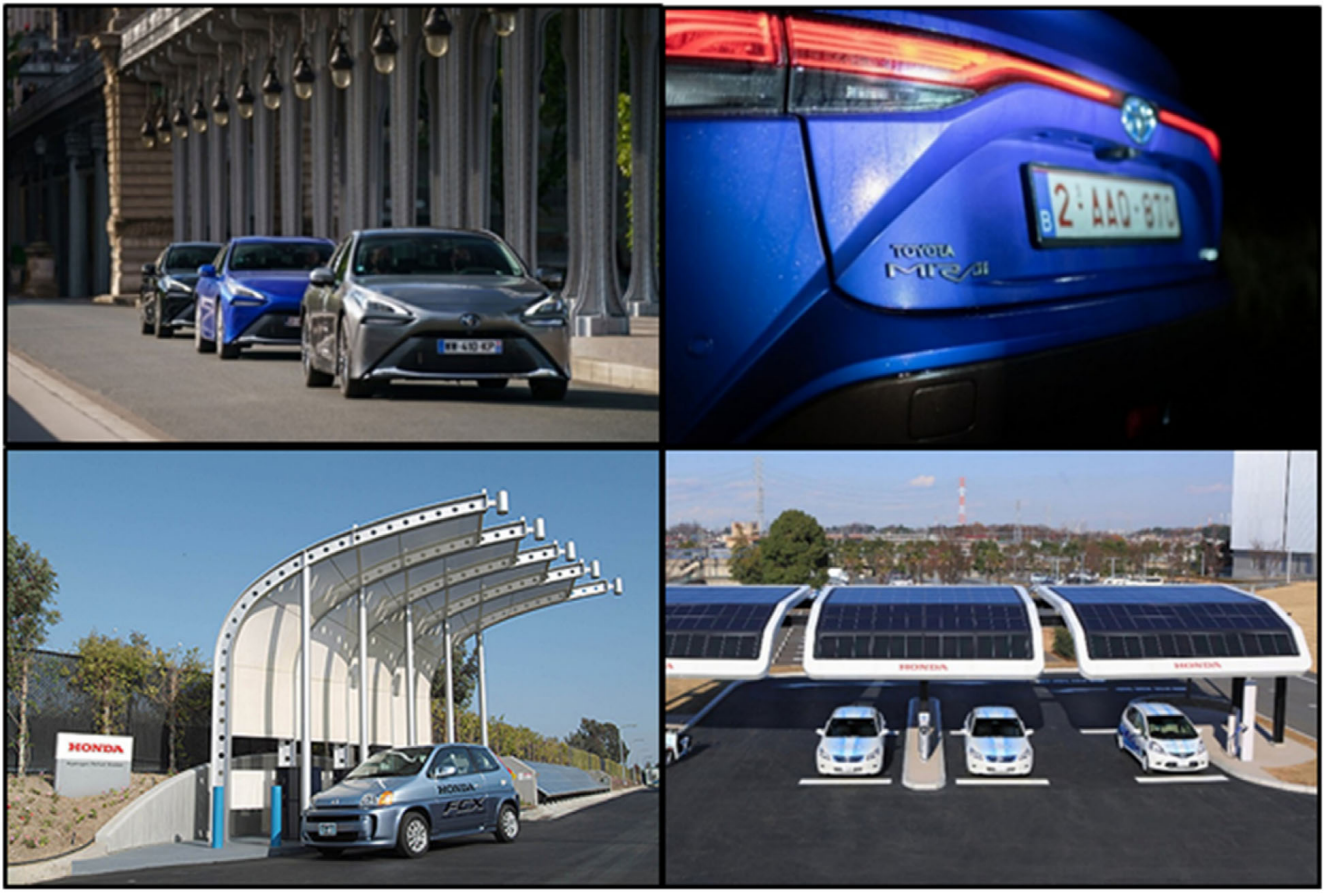
Top panel: In 2021, the Toyota‐developed Mirai model's driving range was 1003 kilometers. Bottom panel: Honda solar photovoltaic‐powered hydrogen fueling facilities Reproduced with permission from ref. [162] Copyright 2023 Chemical society RSC.

Hydrogen fuel cell electric vehicles (HFCEVs) are emerging as a viable alternative in transportation. HFCEVs utilize hydrogen as a fuel source to produce electricity via a chemical reaction within the fuel cell stack. Companies such as Toyota, Hyundai, and Honda have commercialized these vehicles (**Figure** **14**). Recently, Toyota showcased a significant advancement with their Mirai model, utilizing hydrogen as fuel in compact passenger vehicles.^[^ ^174^ ^]^ This vehicle produces no carbon emissions and can travel 650 km (406 miles) using a 5.6 kg compressed hydrogen tank, with refueling possible in 3 min when infrastructure is accessible.

Honda has commercialized renewable hydrogen gas through a solar hydrogen 37 igno cell station specifically designed for its fuel cell vehicles.^[^ ^175^ ^]^ In this scenario, solar photovoltaic (PV) panels provided energy for water electrolysis, resulting in the production of 0.5 kg of H_2_ over the duration of eight hours, which is stored for potential refueling needs. The solar PV‐based electrolysis system developed by Honda is illustrated in Figure 14 (bottom panel).^[^ ^176^ ^]^ The choice to pre‐cool a hydrogen fuel cell vehicle at a more expensive station is contingent upon the balance between time and cost. While vehicles operating at 10 000 psi necessitate a pre‐cooling temperature of 95 °F for optimal performance, this requirement can be circumvented in a 5000 psi bus. Nonetheless, this convenience incurs a cost, requiring 25 min to refuel at a more economical station.^[^ ^177^ ^]^ Therefore, the more expensive pre‐cooling station may justify the faster fill‐up time. The decision ultimately hinges on personal priorities and the specific requirements of the vehicle.

## 6 Challenges in Hydrogen Production Techniques

Both fossil fuels and sustainable energy sources can be used to produce hydrogen; though, there are various methods for producing hydrogen via fossil fuels, including steam reforming, gasification, auto thermal oxidation, and partial oxidation. Wind or solar energy is utilized to split water and gasify bio‐fuels or biomass to make hydrogen.^[^ ^108^, ^109^, ^110^ ^]^ Most of the hydrogen is extorted from coal at an approximate rate of 21.5 billion tones over a year, which must be substituted by sustainable energy resources. Besides the predicted requirement for hydrogen as a portable electricity and transportation fuel, H_2_ will be in high demand due to the heavy oil upgrading, production of ammonia, and upgrading and desulfurization of regular petroleum. Consequently, greater H_2_ generation with current expertise, more conventional hydrocarbons will be required, raising the emission of greenhouse gases. With no net or minimal emission of greenhouse gas (without using carbon sequestration processes), generating hydrogen from renewable sources produced from waste streams or agricultural waste raises the economics and flexibility of semi‐centralized and distributed improvement.^[^ ^111^ ^]^ It is easy to transfer thermocatalytic, biological invention, and electrolysis to on‐site decentralized hydrogen production, eliminating the need for a large and costly distribution infrastructure. Technical obstacles exist with each of these hydrogen manufacturing systems.^[^ ^178^, ^179^ ^]^ These tasks comprise feedstock kinds and exchange efficiency and the requirement to safely integrate hydrogen production systems with H_2_ storage and purification technology.

## 7 Recommendation and Conclusions

Further research is needed into the commercialization of renewable energy‐powered hydrogen manufacturing methods, as well as infrastructure and market development. Water electrolysis systems powered by wind turbines or solar off‐grid PV can also be employed in remote areas without access to the grid. Hydrogen may potentially play an important role in decarburizing the marine industry by providing clean fuel through the utilization of wind energy (offshore). Hydrogen blue can be transported worldwide in the form of ammonia, which easily breaks down on‐site to produce hydrogen. Viable adoption of carbon dioxide exclusion technology can help to achieve net‐zero CO_2_ emissions and prevent global warming near 1.5 °C. Besides its conventional usage as an industrialized raw material for the production of methanol and ammonia, hydrogen is currently being researched as a novel energy source. This paper covers the technologies utilized to manufacture hydrogen from renewable and conventional energy resources, with the significant challenges encountered during the practical deployment of such systems. Hydrogen is formed using sustainable energy resources like solar, geothermal, wind, biomass, hydro, and others. As intermittent sustainable energy sources like the wind and sun are the most capable, hydrogen appears to be the best option for usage as an energy carrier, storage medium, and fuel. Due to the benefits connected with its availability and use of carbon‐free substitutes, hydrogen is obtaining popularity as a potential fuel and exceptional energy transporter choice on a worldwide scale. This paper offers a quick overview of several renewable and conventional energy sources for green hydrogen generation systems. Photocatalytic splitting of H_2_O and the photo‐reforming of oxygen substrates or organic biomass compounds are the most practical and uncomplicated methods for producing H_2_. There is a lot of potential for using biomass photo reforming as a sustainable way to produce energy and useful feedstocks. By making use of economies of scale and increased capacity factors, H_2_ production in remote offshore sites can open the door to inexpensive green hydrogen. The design is intricate, requiring consideration of elements including safety, harsh working conditions, transportation and energy costs, and sea obstacles.

Two of the biggest obstacles are maintaining offshore platforms in challenging conditions and creating state‐of‐the‐art electrolysis technology. Another major barrier is the high cost of setting up and maintaining offshore platforms. This can be resolved by looking for economies of scale, developing fresh funding strategies, and identifying cost‐cutting measures such as using off‐the‐shelf components and modular designs. Impact evaluations and mitigation strategies can be used to lessen the influence of offshore platforms on environmental issues. Communicating with regulatory bodies to guarantee adherence to all pertinent rules and regulations and keeping up with any modifications to the regulatory landscape are two ways to address pertinent international and national regulations. It also discusses each way of manufacturing hydrogen from sustainable energy, taking into account the previous efforts. The key points of the review are as follows:

The use of hydrogen energy for many purposes might be advantageous because of its zero or low emissions in the environment. As a result, many methods of hydrogen synthesis and use have been investigated based on their sources of production. This study discusses actual hydrogen generation systems with the concerns associated with them. Hydrogen used as an energy source can assist to safeguard the environment from hazardous emissions, which is the primary topic of this publication. Because of its environmental friendliness, hydrogen energy has the potential to become a renewable energy source that may be used in a wide range of applications. However, producing hydrogen from resources like fossil fuels will be harmful to the atmosphere. As a result of this review, it is advised that for hydrogen generation, nuclear energy and renewable energy be prioritized in order to reduce emissions to the environment. It is also recommended that in the prospect, alternative techniques of creating hydrogen from diverse sustainable sources be considered in order to assess their efficiency.

## Conflict of Interest

The authors declare no conflict of interest.

## Author Contributions

M.W.A. dealt with conceptualization, methodology, writing the original draft, review and editing, supervision, and project administration. N.D. dealt with the literature review, data curation, writing the review and editing, and validation. M.S.A. dealt with investigation, formal analysis, writing the review and editing, and visualization. M.E.A. dealt with data collection, analysis, writing the original draft preparation, and critical review. A.B.A. dealt with the validation, resource management, and writing the review and editing. A.M.A.‐F. dealt with data interpretation, manuscript structuring, and writing the review and editing. S.A.A. dealt with supervision, funding acquisition, manuscript revision, writing the original draft preparation, and critical review. B.A. dealt with writing the review and editing, and critical review. All authors have read and approved the final manuscript. Authors Biography: Dr. Mir Waqas Alam, recognized in 2024 among the Top 2% of scientists globally by Stanford University, is a leading figure in Nano and Functional Material Science. He is currently an Associate Professor in the Department of Physics at King Faisal University, Saudi Arabia. Since joining the university in 2015 as an Assistant Professor, his research excellence and academic leadership earned him promotion to Associate Professor in 2022. Dr. Alam holds a Ph.D. from Toyama University, Japan, where he made significant strides in material science. He worked research scholar at Western Kentucky University, USA, and post‐doctoral fellow at the National Institute of Material Science (NIMS), Japan.
